# Mitochondrial dynamics in health and disease: mechanisms and potential targets

**DOI:** 10.1038/s41392-023-01547-9

**Published:** 2023-09-06

**Authors:** Wen Chen, Huakan Zhao, Yongsheng Li

**Affiliations:** Department of Medical Oncology, Chongqing University Cancer Hospital, Chongqing, 400030 China

**Keywords:** Cancer metabolism, Tumour immunology

## Abstract

Mitochondria are organelles that are able to adjust and respond to different stressors and metabolic needs within a cell, showcasing their plasticity and dynamic nature. These abilities allow them to effectively coordinate various cellular functions. Mitochondrial dynamics refers to the changing process of fission, fusion, mitophagy and transport, which is crucial for optimal function in signal transduction and metabolism. An imbalance in mitochondrial dynamics can disrupt mitochondrial function, leading to abnormal cellular fate, and a range of diseases, including neurodegenerative disorders, metabolic diseases, cardiovascular diseases and cancers. Herein, we review the mechanism of mitochondrial dynamics, and its impacts on cellular function. We also delve into the changes that occur in mitochondrial dynamics during health and disease, and offer novel perspectives on how to target the modulation of mitochondrial dynamics.

## Introduction

Mitochondria (mt) are highly plastic and dynamic organelles that critical for cellular metabolism, stress responses and homeostasis maintenance. As the sites for biochemical processes such as adenosine triphosphate (ATP) production, fatty acid synthesis, intracellular reactive oxygen species (ROS) generation, oxidative phosphorylation (OXPHOS), thermogenesis and calcium (Ca^2+^) homeostasis, mitochondria are considered as the hub of cellular metabolism.^1,2^ Furthermore, signal intermediates generated in mitochondria during metabolism are important for regulating cellular function and phenotype.^3^ For example, mt-ROS are implicated in the signal transduction of inflammatory responses. Once the toll-like receptor (TLR) signaling is activated by lipopolysaccharide (LPS), mitochondria will be recruited to the phagosome and augmented the production of mt-ROS to enhance the antibacterial activity of macrophages.^4^ In addition, dysfunctional mitochondrial are closely interrelated to a range of disease and pathology including metabolic diseases, neurodegenerative disorders and cancers, which are broadly characterized by impaired mitochondrial function.^5,6^

The process of changes in the morphology, quantity and position of mitochondria within eukaryotic cells are defined as mitochondrial dynamics, which are indispensable for proper functions of the cells, e.g., energy production and other pivotal cellular processes including movement, differentiation, cell cycle, senescence and apoptosis.^7^ Dysregulation of mitochondrial dynamics is one key pathogenic mechanisms of a diverse of diseases and pathologies that are characterized by dysfunctional mitochondrial.^8,9^ For example, ischemia cause excessive mitochondrial fission and fragmentation, resulting in cardiomyocyte death.^10,11^ On the contrary, emerging evidence indicates that enhancing mitochondrial fitness by regulating mitochondrial dynamics could prolong life-span and bring beneficial for health.^12–14^ For instance, mitochondrial fission mediated by dynamin-related protein (Drp1) may be a key determinant of insulin resistance; and aerobic exercise intervention restrains the activation of Drp1 and mitochondrial fission, which improves fat acid oxidation (FAO) and insulin sensitivity.^15^

As such, comprehensive understanding of mitochondrial dynamics will conduce to develop more precise strategies for targeted regulation of mitochondrial function. Herein, we introduce the regulation mechanism of mitochondrial dynamics, its role in mediating cellular function, its alterations in health and diseases, and provide new insights for targeted modulation of mitochondrial dynamics.

## Overview of mitochondrial dynamics

**Fig. 1:**
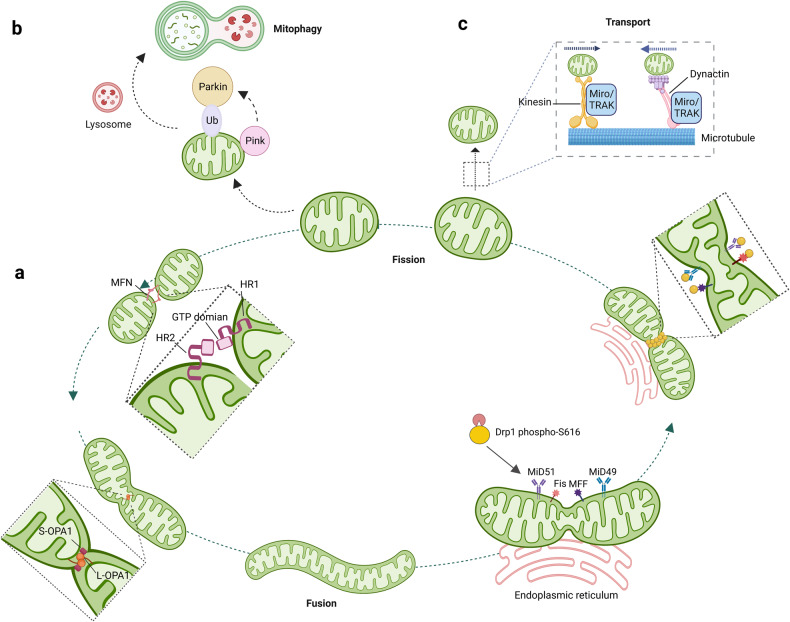
Schematic illustration of the mitochondrial dynamics. **a** mitochondrial fission and fusion. The primary fusion factors involved are Opa1, MFN1, and MFN2, which bind to the inner and outer membranes of mitochondria (IMM and OMM). Fission is mainly mediated by Drp1, which binds to the OMM and forms a ring-like structure around the organelle, resulting in its division into two separate ones. **b** Mitophagy: the PINK/parkin target damaged mitochondria to the lysosome for degradation. **c** Mitochondrial transport along microtubules is facilitated by TRAK/Miro motor adapter complex. Drp1: dynamin-related protein 1; MFN1/2: mitogenic protein 1/2; Opa1: optic atrophy protein 1; Fis1: protein fission 1; Mff: mitochondrial fission factor, PINK: PTEN-induced kinase 1, Miro: mitochondrial rho GTPase

**Fig. 2:**
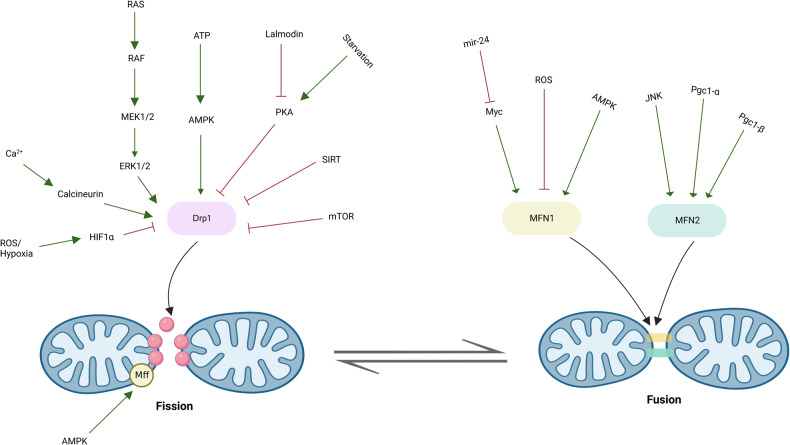
Key proteins and signaling pathways orchestrate mitochondrial fusion and fission. Representative signaling pathways involved in mitochondrial fusion and fission. Green arrows represent stimulation or activation of pathway; red lines represent repression or inactivation of pathway

Mitochondria undergo the continuous processes of fission, fusion, mitophagy and transport cycles, which determine the morphology, quality, quantity and distribution of mitochondria within cells, as well as the mitochondrial function (Fig. 1). Mitochondria have their own DNA and need to constantly repair and replace their components to function properly. Through mitochondrial dynamics, damaged components can be removed from a mitochondrion, or impaired mitochondria can be entirely eliminated by mitophagy, to prevent further cellular damage.^16^ Maintaining a balance of mitochondrial dynamics is pivotal for optimal function of mitochondria and cell fate.^17^ In brief, fission contributes to mitochondrial quality control by elimination of impaired or dysfunctional mitochondria, and promotes apoptosis facing the severe cellular stress, while fusion is conducive to mix and exchange the intramitochondrial contents between mitochondria, these help to maintain mitochondrial function.^16^ Besides, mitophagy is indispensable for quality control of mitochondrial and inhibiting pro-apoptotic proteins release by selective clearance of damaged mitochondria.^18^ Mitochondrial transport is also important for ensuring that mitochondria are located in the proper cellular regions where their energy-generating functions are needed.^19^ At present, it has been established that each step of mitochondrial dynamics is precisely regulated by upstream signaling cascades. We also summarize the key proteins and its upstream signals that orchestrate the process of mitochondrial fission and fusion (Fig. 2).

### Structure of mitochondria

Mitochondria are organelles that enclosed by a double membrane consisting of outer membrane, inner membrane, the inter-membrane space (IMS) and the matrix.^20^ The outer mitochondrial membrane (OMM) is a lipid bilayer that encloses the entire mitochondrion. It is composed of various lipids and proteins that are crucial for its structure and function. The main lipid components of the OMM are phospholipids, which make up approximately two-thirds of the membrane’s total lipid content.^21^ One of the primary functions of the OMM is acting as a barrier between the mitochondria and the cytoplasm. Another important function of OMM is mediating the transport of metabolites and ions in or out of mitochondria. Especially, the voltage-dependent anion channels (VDAC), the major channel of the OMM, plays a pivotal role in transporting metabolic molecules such as ATP, adenosine diphosphate (ADP), and other respiratory substrates across the OMM.^22^ In addition to its structural and transport functions, the OMM is also involved in a variety of intracellular signaling pathways. For example, intrinsic apoptosis pathway activates the pro-apoptotic BCL-2 family proteins Bax/Bak activity, promoting pore formation in OMM allowing cytochrome c (Cyt C) to release from the mitochondria into the cytosol, then activates caspases and initiates apoptosis.^23^ Finally, the OMM carries all the proteins and molecules involved in mitochondrial dynamics.^24^

The inner mitochondrial membrane (IMM) is a highly convoluted membrane that forms invaginations that extend deeply into the matrix called cristae. These cristae provide more surface area and room for matrix, allowing for more efficient OXPHOS.^20^ The most important of these functions is the production of ATP. The electron transport chain (ETC) complexes I-IV integrated into the IMM, transfer electrons from reduced substrates, such as icotinamide adenine dinucleotide (NADH), flavin adenine dinucleotide (FADH2) and Cyt C, to molecular oxygen.^20^ Besides, the IMM is also critical for Ca^2+^ homeostasis. The IMM contains several Ca^2+^ transporters, including mitochondrial calcium uniporter (MCU) and mitochondrial Na^+^/Ca^2+^ exchanger (NCX).^25^ These transporters regulate the flux of calcium ions into and out of the mitochondria, maintaining appropriate calcium levels within the organelle. Generation of ROS is another important function of the IMM.^26^ The IMM carries several enzymes that control ROS production, including superoxide dismutase (SOD), which catalyze the dismutation of superoxide radicals (O_2_^-^) into molecular oxygen and hydrogen peroxide, and glutathione peroxidase (GPX), which catalyzes the reduction of hydrogen peroxide to water and oxygen. Finally, the IMM also plays an important role in the regulation of apoptosis.^27^ The mitochondrial permeability transition pore (mPTP), non-specific channel assembled by a large protein complex that located in the IMM, is involved in the regulation of apoptosis. Opening the mPTP leads to the release of Cyt C and other apoptotic molecules from the mitochondria into cytoplasm, triggering the apoptotic cascades.

The IMS is the smallest and most constricted part of the mitochondria.^20,28^ IMS serves various functions, making it one of the most important sub-compartments in the mitochondria. IMS possesses a wide variety of protein import pathways, which allows the importation of diverse proteins essential in mitochondrial DNA (mtDNA) maintenance, apoptosis, and protein folding.^28^ The OMM sorts and translocates proteins and assembly machinery complex. Once interacted with the protein, mitochondrial IMS imports, and assembly machinery system assists in the insertion of conserved cysteine motifs-containing proteins into IMS.^28^ Additionally, enzymes present in the space are responsible for catalyzing the formation of disulfide bonds, which are pivotal for correct folding and stability of many mitochondrial and secretory proteins.^28^ Furthermore, the IMS regulates cell signaling by controlling calcium signaling and ROS production,^29^ and also plays an important role in the intrinsic apoptosis process, resulting in programmed cell death.^30^ Overall, the IMS is a crucial and multifaceted sub-compartment that contributes to various cellular processes. Any disruption to the IMS homeostasis can lead to mitochondrial dysfunction, which can cause severe health complications such as metabolic disorders, immune system dysfunction, and neurodegenerative diseases.^31^

The space enclosed by the IMM is named as the mitochondrial matrix, which are filled with a fluid containing various metabolic products, enzymes, ribosomes, proteins, as well as mtDNA. Its peculiar structure and composition provide an excellent site for virous biochemical reactions, such as protein biosynthesis, lipid biosynthesis, Krebs cycle, OXPHOS, and mtDNA replication. The main function of the matrix is to produce ATP through OXPHOS by forming a proton motive force across the IMM.^20^ In the matrix, the catabolism of carbohydrates, lipids, and proteins occurs *via* the tricarboxylic acid (TCA) cycle and subsequent OXPHOS, resulting in ATP production.^32^ Apart from energy production, the matrix regulates metabolic processes, e.g., transporting proteins to the mitochondria, maintaining ion balance, and removing ROS. The matrix contains specific transporters, such as the mitochondrial pyruvate carriers^33^ and the ATP/ADP translocases,^34^ that enable molecules to mobilize in and out of the mitochondria. Additionally, the matrix houses chaperones and proteases that monitor protein folding, assembly, quality control, and degradation.^35^ The matrix’s unique composition enables its efficient function in the production of cellular energy, metabolic regulation, and other essential cellular functions.

### Mitochondrial fission

Mitochondrial fission is a multi-step procedure that giving rise to the splitting of one mitochondrion into two.^36^ The process of division primarily occurs at sites where the OMM constricts due to the polymerization of actin or interaction with the endoplasmic reticulum (ER). This process involves the recruitment of the GTPase enzyme Drp1 by various OMM adapter proteins, including mitochondrial fission 1 (Fis1), mitochondrial fission factor (Mff), mitochondrial dynamics protein (MiD)-49, and MiD51.^37,38^ Then, highly oligomerized of Drp1 is activated by mitochondrial specific lipid cardiolipin to form large helical structures and augments GTPase activity at the mitochondrial fission foci.^37^ Nucleotide-driven allostery of Drp1 facilitates its self-assembly, conformational transformation and disassembly to encircle mitochondria and induces mitochondrial fission.

Drp1 exists in the cytoplasm when inactivated, and its activation is regulated by phosphorylation, SUMOylation, ubiquitination and S-nitrosylation modification.^39,40^ Phosphorylation and SUMOylation also regulate mitochondrial recruitment of cytosolic Drp1.^41,42^ During cell mitosis, activation of Drp1 through phosphorylation at S616 by cyclin dependent kinase (Cdk)-1 and protein kinase C isoform delta (PKCδ) facilitates mitochondrial division.^43^ Other kinases, such as activation of mitogen-activated protein kinase (MAPK), extracellular signal-regulated kinase (ERK), Cdk5 and Ca^2+^/calmodulin dependent protein kinase II (CAMKII), have also been reported to phosphorylate Drp1-S616 to facilitate mitochondrial fission.^39^ Additionally, the activity of Drp1 is restrained when protein kinase A (PKA)-mediated phosphorylation of Drp1 at S637, while dephosphorylation at S637 by calcineurin-mediated pathways facilitate mitochondrial cleavage by recruiting Drp1 to the OMM.^44^ On the other hand, phosphorylation of Drp1-S637 by CaMK1α and Rho-associated coiled-coil containing protein kinase 1 (ROCK1) increases the division activity of Drp1, suggesting that except from the phosphorylated site, the cellular context also perform a profound influence on the activity of Drp1.^45^

The activity of Drp1 is also affected by its SUMOylation status. The SUMOylation of Drp1 by SUMO-1 intercepts the lysosomal degradation of Drp1, further promoting mitochondrial division. Conversely, DeSUMOylation of SUMO-2/3 from Drp1 by the enzyme SUMO specific peptidase 3 (SENP3) reinforces mitochondrial fission *via* facilitating interaction of Drp1 with the OMM resident adapter protein Mff.^46^ In addition, AMP-activated protein kinase (AMPK) phosphorylates Mff, then promote the binding of Drp1 with Mff, which facilitates recruitment of Drp1 from the cytosol to mitochondria under energetic stress.^47^ Moreover, E3 ubiquitin ligase MARCH5 mediated-ubiquitination of Drp1 protein and its receptor MiD49 facilitates the degradation of these two protein, therefore suppressing mitochondrial fission.^48^ Finally, S-nitrosylation of Drp1 at C-terminal GTPase effector domain caused by β-amyloid protein facilitates Drp1 assembly and GTPase capability, which triggering mitochondrial fission in Alzheimer’s disease.^49^ Besides, Post-translational modification of Drp1 receptors also affects their stability and activity. For example, AMPK-mediated phosphorylation of Mff promotes Drp1 recruitment to OMM;^47,50^ MiD49 can be ubiquitinated by MARCH5/MITOL and subsequently degraded by proteasome, which restraining mitochondrial fission.^48^ Hence, multiple signaling pathways integrate different post-translational modifications of Drp1 and its receptors to orchestrate the process of mitochondrial fission.

Inhibition of Drp1 activity by dominant-inactivation mutations results in the formation of elongated tangles and collapses of mitochondria.^38^ The knockout of mouse Drp1 through genetic engineering results in embryonic lethality, indicating the crucial role of Drp1-dependent mitochondrial division in embryogenesis.^51–53^ Beyond affecting mitochondrial function and morphology, mitochondrial fission possess other functions, such as promoting mitochondrial transport, mitophagy, cell division as well as apoptosis.^51–53^

### Mitochondrial fusion

Mitochondrial fusion includes several steps, starting with the activation of dynein-associated GTPases, including mitofusin (MFN) 1/2 on OMM, FAM73a/FAM73b and optic atrophy protein 1 (Opa1) on IMM,^54–56^ followed by OMM fusion induced by GTP hydrolysis, subsequently IMM fusion and finally the mixing of intra-mitochondrial components.^54^ Mitochondrial fusion dilutes dysfunctional proteins and mutated mtDNA by mixing mitochondrial proteins, mtDNA and other matrix components to maintain mitochondrial homogeneity and functional stability.^55^ Genetic knockout of MFN1 and/or MFN2, destroys the mitochondrial structure and induces serious cellular defects, including smaller and more fragmented mitochondria, lower mitochondrial membrane potential, attenuated respiration activity and ATP production, which in turn suppresses cell proliferation.^57^

The most efficient process of mitochondrial fusion is observed when both MFN proteins are present simultaneously. Additionally, a deficiency in MFN1 leads to a significantly fragmented mitochondrial morphology, while cells lacking MFN2 display a high percentage (85%) of mitochondria appearing as spheres or ovals, indicating that the functions of MFN1 and MFN2 are distinct.^58^ Importantly, the turnover and activity of MFNs are also precisely orchestrated by post-translational modifications. For example, the acetylation of MFN1 at K222 or K491 inhibits the MFN1 GTPase activity.^59^ In addition, the pro-fusion activity of MFN1 is affected by phosphorylation at multiple sites. For instance, phosphorylation of MFN1 at T562 regulated by MEK/ERK cascade restricts MFN1 assembly and pro-fusion capability, and facilitates its interaction with the proapoptotic protein Bak, leading to impaired mitochondrial fusion and apoptosis.^57^ Under glucose deprivation, histone deacetylase 6 (HDAC6)-mediated deacetylation of MFN1 at K222 or K491 causes increased MFN1 activity and hyperfused mitochondrial networks.^59^ Furthermore, PRKN-dependent ubiquitination and proteasomal degradation of MFN2 is involved in OMM-ER contact site remodeling and the enhanced mitophagy.^60^ Under cellular stress, MFN2 is phosphorylated at Ser27 by Jun N-terminal kinase (JNK), and subsequently degradated by the proteasome, which is mediated by E3-ubiqutin ligase HUWE1.^61^ Intercepting the phosphorylation at Ser27 reduces MFN2 degradation, facilitates mitochondrial elongation, and prevent apoptosis. Hence, mitochondrial dynamics are integrated into various physiological activities and signaling cascades through regulating the post-translational modifications of MFNs.

On the other hand, transcriptional regulation of MFNs also affects mitochondrial fusion in response to stress conditions. The transcriptional coactivator peroxisome proliferator-activated receptor gamma coactivator 1 (PGC1)-β upregulates MFN2 expression through transcriptional activation, thereby promoting mitochondrial fusion and OXPHOS.^62^ Besides, MFN1 is identified as a target for miR-140, and miR-140 negatively regulates the transcriptional expression of MFN1, thereby promoting mitochondrial fission and apoptosis.^63^

Fusion of IMM is mainly regulated by the optic atrophy 1 (Opa1), which is a dynamin-like GTPase inserted into the IMM by its N-terminal.^64^ There are two splicing forms of Opa1: IMM-anchored long form-Opa1 (L-Opa1) and soluble short form-Opa1 (S-Opa1). L-Opa1 is proteolytically hydrolyzed to S-Opa1 by OMA1 and YME1 Like 1 ATPase (YME1Ll), and the relative levels of L-Opa1 and S-Opa1 are a key factor in determining the viability of mitochondrial fusion.^65^ In addition to controlling the IMM fusion, Opa1 also orchestrates cristae integrity, mtDNA maintenance, bioenergetics, as well as respiratory chain super complex assembly, so that Opa1 directly affects mitochondrial cytochrome release and oxidative respiration efficiency.^51,66^ Early embryonic lethality are observed in double knockout or OPA1 mutant mice.^67^ Furthermore, mutations in Opa1 gene are detected in 60–70% of autosomal dominant optic atrophy (ADOA) patients, characterized by retinal ganglion cells lost along with impaired visual acuity at early age.^68^

### Mitophagy

Mitophagy, an evolutionarily conserved process that selectively removes dysfunctional or superfluous mitochondria by autophagy, is pivotal for both mitochondrial quantity and quality control.^18^ Mitophagy is a complex and dynamic process that involves two steps. Firstly, dysfunctional or damaged areas of mitochondria are identified and selectively enclosed by double-membraned autophagosomes. Secondly, the autophagosomes fuse with lysosomes to form autolysosomes where damaged mitochondria are degraded by hydrolases. At present, the pathway comprised of PTEN-induced kinase 1 (PINK1) and Parkin, an E3 ubiquitin ligase, is identified as a key player of mitophagy in mammals.^69^ In healthy mitochondrial, PINK1 is rapidly cleaved, and subsequently degraded in a proteasome-dependent manner in IMM. In damaged mitochondria, IMM depolarization prevents the degradation of PINK1, causing accumulation of full-length PINK1 with kinase activity in the OMM. Then, PINK1 recruits parkin to the OMM and stimulates its ubiquitin ligase activity *via* phosphorylating ubiquitin at Ser65. Parkin-mediated ubiquitination facilitates the degradation of multiple OMM proteins, such as MFN1, MFN2, and VDAC1, while also attracting autophagy receptors such as p62 and optineurin. Consequently, the ubiquitinated mitochondria are combined with LC3-positive autophagosomes by these receptors, leading to the formation of autophagosomes that eliminate damaged mitochondria.^69,70^ Impaired mitophagy refers to the inability of cells to effectively eliminate dysfunctional mitochondria, leading to their accumulation and disruption of mitochondrial function and cellular homeostasis. This phenomenon is closely associated with a variety of diseases, including neurodegenerative and cardiovascular diseases.^70^

### Mitochondrial transport

Spatial distribution of mitochondria regulated by mitochondrial transport has been proved to be critical for highly polarized cells including neurons.^71^ Neurons are consisted of three distinct regions: soma, long axon and thick dendrites. Axonal transport, mitochondrial movement from soma to distal axonal, is driven by microtubule-anchored kinesin1 (also called KIF5) and dynein motors, while etrograde movement (toward soma) of mitochondria is orchestrated by cytoplasmic dynein‐dynactin complex.^72^ Furthermore, these opposing microtubule-based motors are linked to mitochondria through interaction with TRAK family adapter proteins including TRAK1 and TRAK2, and mitochondrial rho (MIRO).^73^ The direction of mitochondrial movement within the cell is determined by the balance of forces between motor and anchor proteins, both simultaneously present on the OMM of one specific mitochondria.^74^ Mitochondrial dysfunction triggered by aberrant mitochondrial transport is identified as one of the important pathogenic factors of neurodegenerative disorders and cancers.^72,74^

## Mitochondrial dynamics and cellular function

**Fig. 3:**
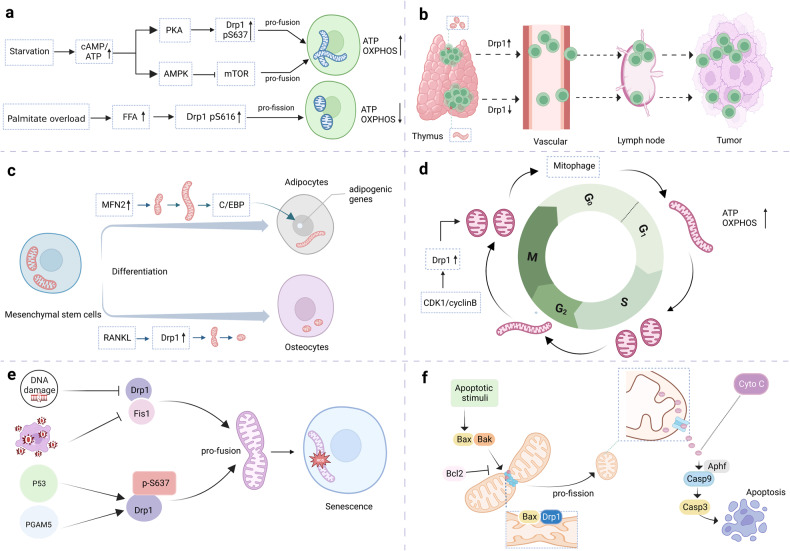
Mitochondrial dynamics and cellular function. **a** Mitochondrial dynamics and cell metabolism under different nutrient supplies. **b** Mitochondrial dynamics in the movement of mature circulating T cells. **c** Mitochondrial dynamics in cell differentiation. **d** Mitochondrial dynamics in cell cycle. **e** Mitochondrial dynamics in cellular senescence. **f** Mitochondrial dynamics in cellular apoptosis

As important signaling organelles, mitochondria adjust a series of functions in cell including cellular metabolism, energy production and ion homeostasis, senescence and apoptosis, which dictate the fate of cells. Many mechanisms aid to the transmission of mitochondrial fitness to cells. Emerging studies shows that mitochondrial dynamics are important contributors to diverse cellular function (Fig. 3).

### Cell Metabolism

Literature has revealed a fascinating correlation between the balance of energy demand and supply, and the structure of mitochondria. When cells are grown in nutrient-rich environments, they often exhibit fragmented mitochondria with impaired OXPHOS, reduced mtDNA, and increased production of ROS. On the other hand, mitochondria in cells experiencing nutritional deficiencies tend to remain connected for longer periods, maintaining highly efficient OXPHOS and increased levels of ATP.^75^

Under low nutrient supplement, several metabolic sensor kinases can drive mitochondrial elongation.^76^ PKA, one of the kinases regulating phosphorylation at the Ser637, acts in a cAMP-dependent manner which is indirectly responsive to high and low changes in cAMP/ATP.^76,77^ In a similar manner, activation of AMPK or restrain of mammalian target of rapamycin (mTOR) is demonstrated to induce mitochondrial fusion during nutrient depletion in cultured cells.^76^ These findings suggest that bioenergetic adaptation, involving changes in bioenergetic efficiency and ATP synthesis capacity, is associated with the remodeling of mitochondrial architecture.

In turn, mitochondrial dynamics also orchestrates the cellular metabolism.^78,79^ Mitochondrial fission caused by deficiency of MFNs or decline in GTPase activities manifests as metabolic dysfunction such as suppression of OXPHOS, decreased ATP synthesis, mtDNA depletion and elevated ROS levels, which are involved in various pathological conditions. Alternatively, the fusion of mitochondria counteracts metabolic insults primarily through enhancing OXPHOS and ATP generation, as well as by preventing mitochondria against mitophagy.

Mitochondrial dynamics also possesses the capacity to regulate cellular metabolism in immune cells, thereby influencing the activation state and function of these cells. For instance, macrophages will employ glycolysis exclusively when they are polarized to a pro-inflammatory state known as M1, whereas macrophages in M2 state, which play a pivotal role in healing wounds and repairing tissues, depend on increased OXPHOS.^80^ Inhibiting mitochondrial fission in LPS-activated macrophages with Mdivi-1, an antiDrp1 agonist, effectively reduces glycolytic reprogramming.^81^ In addition, morphological analysis also demonstrates that mitochondria exhibit distinct shapes in order to assist in the differentiation of T cells.^81^ Once Drp1 is phosphorylated at Ser616, effector T cells exhibit higher mitochondrial fission frequency. As a result, the mitochondria of these cells are smaller and scattered throughout the cytoplasm, facilitating anabolism. Unlike effector T cells, memory T cells depend on catabolic metabolism for their prolonged survival, which show an increased presence of fused mitochondria. The distinction in mitochondrial morphology among memory T and effector T cells delineates their differential metabolic requirements.^81^ When mitochondrial fusion is inhibited through selective removal of Opa1 from T cells, it leads to decreased OXPHOS and alterations in the structure of the cristae.^81^ Together, the stability of the fragmentation and merging mechanisms within mitochondria determines cellular metabolism and is critical for cell function.

### Cell movement

Cell movement is fundamental for several different facets of physiological function, including tissue growth, healing of wounds, immune defense, and for disease-related processes including malignant metastasis. Mitochondria are highly polarized towards the apical portion of migratory cells, where they provide energy for actin cytoskeleton remodeling.^82^ In addition, mitochondrial fission and fusion events at the leading edge are crucial for localized calcium signaling, which is necessary for directional migration.^83^ Moreover, variations occurring in mitochondrial dynamics may also have an influence on various types of cellular movement, including epithelial-mesenchymal transition (EMT),^84^ metastasis,^85^ and trafficking of immune cells.^86,87^

When epithelial cells undergo EMT, they lose their cell-cell junctions and polarization, acquiring the phenotype of mesenchyme.^88^ As a result, cancer cells are able to invade and metastasize more easily. In hepatocytes, increased mitochondrial fission caused by low expression of PGC-1α promoted EMT.^84^ Similarly, loss of GCN5L1 facilitates ROS generation through reinforcing FAO, activating ERK and Drp1, which then initiates mitochondrial rupture, ultimately causing hepatocellular carcinoma (HCC) EMT and metastasis.^89^ Metastatic breast cancer cells have been found to exhibit a higher concentration of fragmented mitochondria, along with increased levels of Drp1 and fewer MFN1 molecules compared to nonmetastatic breast carcinoma cells. The overexpression of MFN1 or silencing of Drp1 leads to mitochondrial elongation or aggregation, which in turn significantly reduces the metastasis of breast cancer cells.^85^

Immune cell transport involves various aspects such as immune cell migration, infiltration, and homing, which is also adjusted by altered mitochondrial dynamics. For example, in T lymphocytes, mitochondrial splitting assists in the formation of so-called leading edges, which determines cell movement and migration.^87^ Besides, inhibiting mitochondrial fission during T cell development leads to a decrease in the population of T cells with maturity within the thymus, and impedes the migration of these cells to the next station lymphoid organ.^87^

### Cell differentiation

Cellular differentiation is the process by which stem cells are transformed into specialized cell types with unique functional properties. Cell differentiation is regulated by multiple signal transduction pathways and transcriptional modulation mechanisms. According to new scientific findings, mitochondrial dynamics influence the process of cell differentiation.^90^

Mesenchymal stem cells (MSCs), which originate in various connective tissues, are versatile stromal cells capable of differentiating into diverse cell lineages, including osteoblasts, adipocytes, and myoblasts. Metabolic activity patterns in MSCs are different from those in their differentiated progeny, with MSCs exhibiting a greater prevalence of the glycolytic pathway, although differentiating cells are more likely to be reliant upon OXPHOS.^91^ During adipogenesis, the process of differentiation of MSCs into adipocytes, there is a switch in mitochondrial dynamics has occurred from fission to fusion.^92,93^ Early in the process of adipogenesis, MFN2 is highly expressed, altering the process towards mitochondrial interfusion.^92^ As a result of the elongated mitochondria, ATP is produced, which activates CCAAT/enhancer-binding protein (C/EBP), ultimately causing genes involved in adipogenesis to be expressed, including C/EBPα, adiponectin, and peroxisome proliferator-activated receptor gamma (PPARγ).

In contrast, high rate of generation of small, fragmented mitochondria is observed as the MSCs differentiate into osteoblasts, which indicates more mitochondrial fission in osteogenesis.^92^ Additionally, a study has demonstrated that mitochondrial rupture triggered by the NF-κB ligand (RANKL)/GSK-3β/Drp1 axis contributes significantly to osteoblast differentiation.^94^ Downregulation of Drp1 leads to impaired osteoclast differentiation and attenuates the condition in a mouse model of postmenopausal osteoporosis.^94^ Based on these findings, it appears that abnormal mitochondrial dynamics may assist to the development of postmenopausal osteoporosis.

Besides, mitochondrial dynamics play a crucial role in the differentiation process of immune cells. As naive T cells differentiate into effector T cells, their mitochondrial mass and ATP production increase, which is essential for the energy-intensive process of activating the immune response and producing cytokines.^95^ Effector T cells formation requires the activation of mitochondrial biogenesis pathways that promote the synthesis of new mitochondria essential for enhanced cellular metabolism.^95^ Alternatively, Tregs possess more fused mitochondria, which enhances their capacity to produce ATP.^96^ The unique properties of elongated mitochondria in Tregs are critical for their immunosuppressive function and maintaining tissue homeostasis.

### Cell cycle

During cell cycle progression, the morphology, distribution, and function of mitochondria undergo dynamic alteration.^97,98^ The cell is in the G1 phase of its division, mitochondria engage in the process of fusion, resulting in the creation of an extensive, interconnected network of tubules that are responsible for facilitating OXPHOS and generating ATP.^98^ As cells progress into the S phase, mitochondria undergo fission to create smaller, more mobile mitochondria that can be distributed to daughter cells during mitosis.^99^ Of note, during mitosis, fission helps to selectively remove damaged or dysfunctional mitochondria. This process ensures that only healthy mitochondria are passed on to daughter cells, reducing the risk of oxidative stress and genomic instability.

Drp1 is involved in the cell cycle. Deficiency of Drp1 causes the abnormal mitochondrial networks distribution around the microtubule organizing center. This aberrant distribution leads to chromosomal instability, excessive centromeric replication, aberrant mitotic spindles, replication stress, and arrest during the G2/M phase.^100^ Furthermore, cyclins and CDKs,^101^ and mitotic regulators (such as the Aurora family of kinases) also regulate mitochondrial dynamics.^102^ In the early M phase, the phosphorylation of Ser616 by CDK1/cyclin B within human Drp1 promotes desintegration of mitochondria.^101^ Aurora A, an upstream of CDK1/cyclin B, targets Drp1 by phosphorylating RALA. This phosphorylated RALA is then released from the cytoplasmic membrane and accumulates on the mitochondrial surface along with its effector protein. As a result of this binding, RALBP1 binds to CDK1/cyclin B, activating its kinase activity and leading to the phosphorylation of Drp1 at Ser616.^102^ This means that an absence of either RALA or RALBP1 results in dysfunctional mitochondrial separation and diminished cell numbers during mitosis.

### Senescence

Senescence is the result of cellular stress factors such as oxidative stress, telomere shortening, and DNA damage causing permanent growth arrest.^103^ Senescent cells are characterized by a number of changes, including alterations in cell morphology and function. An important feature of senescent cells is the development of large, elongated mitochondria.^104^ A possible explanation for the observed phenomenon may be related to variations of gene expression patterns of key mitochondrial fragmentation and merging regulators. According to recent research, senescent endothelial cells in humans exhibit long interconnected mitochondria in response to decreased expressions of Fis1 and Drp1.^105^ Deferoxamine (DFO) is an iron chelating agent that induces senescence phenotype in cultured cells,^106^ making it an effective tool for analyzing the phenomenon of mitochondrial prolongation during cell aging. As a result of DFO-induced senescence in Chang cells, elongated giant mitochondria are formed.^107^ The process of forming giant mitochondria is linked to an increased fusion process and a decrease in the expression of Fis1 during DFO-induced senescence. Conversely, overexpression of Fis1 can reverse both the mitochondrial lengthening and senescence phenotypes induced by DFO.^107^

Senescence mediators, such as p53, affect mitochondrial dynamics by promoting highly interconnected and elongated mitochondrial formation prior to inducing cellular senescence.^108^ According to the mechanism involved, the expression of p53 was followed by the accumulation of inhibitory Drp1 phosphorylation at the Ser637 site, which in turn inhibited Drp1’s translocation to mitochondria.^108^ Furthermore, the IMM serine/threonine phosphatase, phosphoglycerate mutase family member 5 (PGAM5), is a critical component of mitochondrial fission because it dephosphorylates Drp1 at Ser637, which is essential to mitochondrial fission.^109^ PGAM5 deletion results in more fused mitochondria, decreased turnover of mitochondrial, greater ATP and ROS production, and enhanced mTOR and interferon regulatory factor/IFN signaling, and ultimately cellular senescence.^110^

### Apoptosis

Apoptosis, a mechanism for programmed cell death that is critical for mammalian development, as well as serving as a fundamental process for cellular homeostasis and defense against infections. Apoptosis is governed primarily through two groups of proteins: family Bcl-2, containing elements that are either proapoptotic (e.g., Bak and Bax), or antiapoptotic (e.g., BCL-2) members, which initially trigger the process; and the caspase family, whose members are responsible for the execution phase.^111,112^ The Bcl-2 protein family plays a crucial role in regulating the release of proapoptotic molecules from the IMS to the cytoplasm and maintaining the integrity of the OMM.^113^ Although the exact mechanism is not fully understood, the antiapoptotic factors of the Bcl-2 family have prominent roles in stabilizing the barrier capability of the OMM. While proapoptotic proteins like Bak or Bax generally counteract this function and induce permeabilization of the OMM.

Mitochondria play a crucial role during the initiation of the apoptotic phase by secreting proapoptotic molecules, activating caspases, and inducing chromosomal condensation and fragmentation.^114^ An extrinsic as well as an intrinsic pathway can trigger apoptosis. Extrinsic pathway involves no direct interaction with the mitochondria, while the initiation of intrinsic pathway requires Cyt C to be released from the IMS of mitochondria, along with cristae disruption and mitochondrial outer membrane permeabilization (MOMP). Apoptotic protease factor 1 (Apaf-1) was activated by this process, which is an essential step within the intrinsic pathway, resulting in the activation of procaspase-9.^114,115^

The process of apoptosis leads to a dramatic reorganization of mitochondrial networks into punctate spheres rather than long interconnected tubules.^116^ Given that apoptotic cells display a high fission/fusion ratio, it is believed that fission is an essential part of the apoptotic process.^117^ It appears that Drp1 contributes to this fracturing phenotype because previous studies have confirmed that depletion of Drp1 prevents division of mitochondria during apoptosis,^118^ and dominant negative Drp1 (Drp1K38A) overexpression inhibits the fragmentation of mitochondria during apoptosis as well.^119^ During early stages of apoptosis, proapoptotic molecules such as Bax and Bak, which are able to create pores, are moved to specific mitochondrial foci. These foci are then found to be colocalized with MFN2 and Drp1, which ultimately lead to the formation of sites for mitochondrial fission. It is believed that these foci play a role in preventing the merging of mitochondria, resulting in the fragmentation of mitochondria during apoptosis.^120^

However, according to another study, mitochondrial division is not crucial for MOMP and apoptosis in mammalian cells.^121^ Overexpression of fission proteins can induce apoptosis, and this could be minimized by Bcl-2 family antiapoptotics independently of the transition of mitochondrial morphology from tubular to punctate. This suggests that mitochondrial fragmentation and apoptosis may not always be directly associated.^121^ The researchers used photobleaching fluorescence recovery to assess mitochondrial fragmentation and when Bak or Bax were overexpressed, mitochondria were disconnected. While Mcl-1 or Bcl-xL inhibited the appearance of biomarkers for apoptosis such as Smac/DIABLO and Cyt C in these cells, the fragmentation of mitochondria continued, suggesting that mitochondrial fission and MOMP are separate events.^121^

Opa1-dependent cristae remodeling, characterized by the widening of the neck of the cristae, is another feature of changes in mitochondrial morphology related to apoptosis that facilitates Cyt C release.^122^ Knockdown of Opa1 induces mitochondrial division and impaired cristae structure, and increases the vulnerability to apoptosis.^123^ Furthermore, introducing a disassembler-resistant Opa1 Q297V mutant inhibits the release of Cyt C and the onset of apoptosis, whereas it has no influence on Bax activation.^123^ Although mitochondrial fission’s function in caspase activation during apoptosis remains controversial, abundant literature indicates the significance of mitochondrial dynamics, including both division and merging, as core mechanisms of cell death.

Overall, mitochondrial dynamics play a crucial role in maintaining optimal mitochondrial function, which is essential for energy production and other vital cellular processes.

## The pathophysiology of mitochondrial dynamics

**Fig. 4:**
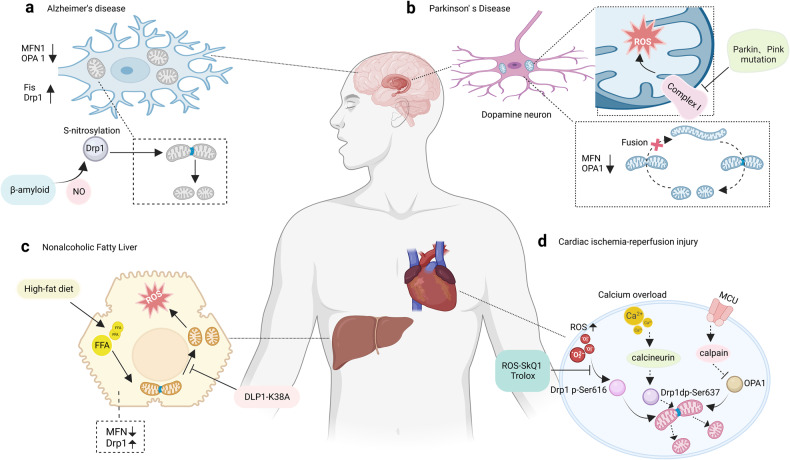
Mitochondrial dynamics and diseases. **a** The neuronal cells of Alzheimer’s disease patients exhibit small and fragmented mitochondria; the expression levels of Opa1, MFN1, and MFN2 were reduced while Fis1 and Drp1 are increased; Beta-amyloid causes nitric oxide to produce and results in neuronal injury and mitochondrial fission by S-nitrosylation of Drp1. **b** Mitochondria in cells with Parkinson’s Disease are also small and fragmented. **c** The progression of non-alcoholic fatty liver disease is closely associated with mitochondrial fission and an increase in protein expression of Drp1. A high-fat diet can cause mitochondrial fragmentation, which occurs prior to the generation of ROS. **d** Ischemia-reperfusion injury can lead to mitochondrial fragmentation through the activation of Drp1, and downregulation of Opa1

The imbalanced mitochondrial dynamics are correlated with a series of diseases which are extensively marked by deficiencies in mitochondrial function and abnormal cellular fate (Table 1; Fig. 4). On the contrary, enhancing mitochondrial fitness by regulating mitochondrial dynamics is proven to reduce the risk of disease, and is beneficial for health.

**Table 1:** Clinical syndromes associated with encoding fission and fusion machinery components

Protein	Disease	Protein Expression	Mitochondrial Morphology	Clinical Outcome/Symptoms	Ref.
**Drp1**	Diabetes	Increased	Fragmentation	Impaired glucose metabolism and insulin resistance,	^197,198^
	Non-alcoholic fatty liver disease	Increased	Fragmentation	Hepatic lipid metabolism, oxidative stress, hepatic damage	^204,205,208,210^
	Parkinson’s Disease, Alzheimer’s Disease, Huntington’s Disease	Increased	Broken cristae, increased fragmentation	Depletion of mitochondria from dendritic spines, decreased ATP production, motor and cognitive impairments	^173,184,191–194^
	Ischemia-Reperfusion Injury	Increased	Excessive mitochondrial fission and fragmentation	Myocardial cell death	^10,11^
	Heart Failure	Increased	Fragmentation	Reduced energy supply to the heart muscle cells, impairing contractile function and worsening HF progression	^221^
	Cardiomyopathy	Increased	Excessive mitochondrial fission	Abnormal cardiac structure and function	^229^
	Various types of cancer	Increased	Fragmentation	Increased cell proliferation, decreased apoptosis, tumor growth, cell migration and invasion	^235,236,240,241,243,244^
**FIS1**	Diabetes	Increased	Fragmentation	Impaired insulin-stimulated glucose uptake	^148^
	Alzheimer’s Disease, Huntington’s Disease	Increased	Fragmentation	Dysfunctional mitochondrial dynamics	^185,191^
**MFN1**	Various types of cancer	Decreased	Fragmentation	Inhibited cell proliferation, invasion, and migration, inhibited metastasis, promoted mitochondrial fusion	^245,247^
	Alzheimer’s Disease, Huntington’s Disease	Decreased	Fragmentation	Dysfunctional mitochondrial dynamics, impaired neuronal transport	^185,191^
	Heart Failure	Decreased	Small and fragmented mitochondria	Impaired ATP production, increased ROS generation	^222^
	Cardiomyopathy	Decreased	Disrupted mitochondrial fusion	Dilated cardiomyopathy	^226^
**MFN2**	Non-alcoholic fatty liver disease	Decreased	Fragmentation	Diminished hepatic function, inflammation, triglyceride accumulation, fibrosis, and hepatic cancer	^208,209^
	Alzheimer’s Disease, Huntington’s Disease	Decreased	Fragmentation	Dysfunctional mitochondrial dynamics, impaired neuronal transport	^185,191^
	Charcot-Marie-Tooth Disease,	Decreased	Fragmentation	Severe progressive muscle weakness, motor deficits and peripheral neuropathy	^160,162^
	Autosomal Dominant Optic Atrophy	Decreased	Fragmentation	Degeneration of the retinal ganglion cells leading to optic atrophy and visual impairment	^166^
**OPA1**	Alzheimer’s Disease, Huntington’s Disease	Decreased	Fragmentation	Dysfunctional mitochondrial dynamics, impaired neuronal transport	^185,191^
	Autosomal Dominant Optic Atrophy	Decreased	Fragmentation	Degeneration of the retinal ganglion cells leading to optic atrophy and visual impairment	^166,167^
	Ischemia-Reperfusion Injury	Decreased	Inactivation and mitochondrial fission	Myocardial cell death	^218^
	Heart Failure	Decreased	Reduced expression and decreased fusion	Impaired ATP production, increased ROS generation	^222^
	Cardiomyopathy	Decreased	Disrupted mitochondrial fusion	Dilated cardiomyopathy	^227^
**MFF**	Cardiomyopathy	Increased	Mitochondrial fragmentation	Dilated cardiomyopathy, heart failure, and death	^231^

### Improved mitochondrial dynamics in health

#### Exercise

Exercise training improves physical performance and provides health benefits by promoting skeletal muscle adaptations, particularly in mitochondrial quantity and quality.^124^ Recent research has shown that exercise training leads to positive outcomes by promoting mitochondrial adaptation, which involves improvements in mitochondrial dynamics and clearance in addition to traditional promotion of mitochondrial biogenesis. John Holloszy’s research in 1967 was groundbreaking as it established that exercise training can promote skeletal muscle mitochondrial biogenesis. In his pioneering study, he demonstrated that vigorous treadmill running in rats induced significant increases in the activity of mitochondrial proteins and enzymes in the recruited muscles.^125^ Subsequent studies utilizing stable isotope techniques have further substantiated these findings by providing definitive evidence that exercise stimulates protein synthesis in mitochondria and biogenesis of mitochondria, in muscle of human skeletal.^126^

AMPK, a critical biosensor of energy, is activated following exercise and promotes the biogenesis of mitochondria by phosphorylating PGC-1α.^127^ PGC-1α stimulates the biological production of mitochondria through the amplification of mitochondrial genes encoding nuclear protein, including those involved in mitochondrial fusion and OXPHOS.^128^ The MAPK signaling pathway is also activated during exercise and promotes the biogenesis of mitochondria *via* augmentation of mitochondrial transcription factor A and PGC-1α, which regulates mDNA transcription and replication.^129^

In addition to inducing mitochondrial biogenesis, exercise also triggers mitophagy through various mechanisms, including PINK1/Parkin and BNIP3/NIX.^130^ The PINK1/Parkin pathway is activated when the mitochondria undergo a depolarizing condition, leading to the recruitment of Parkin under stress and the disposal of damaged mitochondria through autophagy.^130^ On the other hand, the BNIP3/NIX pathway is activated during exercise by hypoxia, which promotes mitochondrial turnover by inducing mitophagy.^131^

Exercise regulates mitochondrial fusion and fission through various pathways. During exercise, as the bioenergetic demand increases, the ratio of AMP/ATP increases as well, a signal that is detected by AMPK.^132^ In response to exercise, AMPK phosphorylates OMM protein Mff and A-kinase anchoring protein 1 (AKAP1), indicating a pro-fission response following exercise.^47,132^ Upon phosphorylation, AKAP1 binds PKA to the OMM, leading to increased PKA activity that causes Drp1 to be phosphorylated at Ser637.^132^ This phosphorylation inhibits the GTPase activity of Drp1,^133^ resulting in an overall pro-fusion effect, and this process is dependent on AMPK.

Acute exercise is potentially connected with mitochondrial fragmentation, possibly due to elevated energy demands during exercise.^134,135^ However, subsequent recovery or exercise training have been shown to be associated with increased mitochondrial volume and connectivity, indicating a possible shift towards improved mitochondrial function and fusion.^15,136^ Recent studies have investigated gene and protein expression patterns associated with fission and fusion resulting from both single exercise sessions and repeated exercise training. These studies aim to gain a deeper understanding of the molecular mechanisms that underlie the changes in mitochondrial dynamics induced by exercise. In particular, MFN1/2 gene transcript expression remains constant 2 hours following a single bout of intensive exercise, but increases at 24 hours afterward. This increase has been attributed to the involvement of transcriptional co-activators that are crucial in the response to exercise, such as PGC-1α and the estrogen receptor, which are known to promote mitochondrial biogenesis.^137^ No significant changes in the protein levels of Fis1, Drp1, or MFN1/2 were observed 4 hours after a high-intensity interval training session in humans. However, an increase in the protein contents of MFN1, Fis1, and Drp1 was observed after a 2-week period.^128^ Several recent studies have also reported similar increases in MFN1/2 concentrations in skeletal muscles following exercise, although not all studies have consistently shown these changes.^134,136^

The study of exercise as a means to improve disease by altering mitochondrial morphology holds immense potential for developing novel therapeutic approaches for various diseases. A study has revealed that physical activity training can reduce the activation of Drp1 on muscle cells in individuals with insulin resistance. This highlights a significant correlation between decreased Drp1 activity and enhancements in both the oxidation of fat and insulin sensitivity following this type of training.^15^

#### Longevity

The decline in mitochondrial function with age, which is accompanied by morphological changes and reduced respiration in various tissues and organisms, is not fully understood in terms of whether it is the initial cause of aging or simply a reflection of aging.^138^ Furthermore, there is a continuing debate regarding the possibility that some of these alterations are caused by mitochondria damage or if they are adaptive mechanisms designed to counteract impairments associated with aging.

In various animal models, researchers have found that the accumulation of mutations in mtDNA and the decrease in mitochondrial biosynthesis can lead to the deterioration of mitochondrial function due to aging.^139,140^ Furthermore, studies conducted on *Caenorhabditis elegans*, yeast, and mice demonstrate that impaired degradation of deficient mitochondria can lead a buildup of unhealthy mitochondria with the aging process.^12,141–143^ In yeast and *C. elegans*, downregulation of mitochondrial autophagy proteins results in mitochondrial damage and a shortening of lifespan.^12,141^ Whereas activating mitochondrial autophagy is linked to improved mitochondrial functionality and increased lifespan in mice and *C. elegans*.^142,143^ Additionally, modifications to mitochondrial dynamics, a mechanism that is crucial in maintaining the quality and functionality of mitochondria, have been implicated in changes in life expectancy in *Drosophila*, yeast, and *C. elegans*.^12–14^

With age, there is a decrease in the protein levels required for mitochondrial fission. For example, aging mice show decreased Drp1 activity as well as morphological changes in mitochondria throughout various tissues, particularly in muscles and neurons.^144^ Similarly, cells from aged human endothelial tissue cultured in vitro also show decreased Fis1 and Drp1 expression, along with elongated mitochondrial networks.^105^ Notably, increasing Drp1 expression in midlife *Drosophila* leads to prolonged lifespan and improved mitochondrial respiration and autophagy.^145^ Additionally, inducing fragmented mitochondria within the intestinal tract has been shown to promote longevity in both *C. elegans* and flies, indicating that maintaining fission of mitochondria may have a beneficial effect on extending life span.^146^

In contrast, studies in yeast have revealed paradoxical results, indicating that eliminating Dmn1p, the ortholog of Drp1, slows down mitochondrial division, actually prevents aging in yeast without affecting fertility or growth rate.^147^ On the other hand, research in skeletal muscle from mice indicates that a rise of mitochondrial fragmentation is related to an impaired insulin response and dysfunctional mitochondria.^148^ Together the available research suggests that the impact of stimulating mitochondrial splitting on mitochondrial function and fitness is dependent on the context, with variations observed based on the type of tissue or organism under study. While the molecular mechanisms and signaling pathways involved in mitochondrial fission and their impact on longevity are not yet fully understood.

Mitochondrial fusion has been found to have an impact on longevity. A study in *C. elegans* shows that an increase in mitochondrial fusion supports the health of aged creatures in various ways.^149^ Insulin/IGF-1 signaling pathway (IIS) and the Cullin-RING ubiquitin ligase (CRL) regulate fusion of mitochondria in *C. elegans*. Specifically, IIS regulates the activity of CAND-1 protein, while the CRL complex SCFLIN-23 regulates mitochondrial fusion through ubiquitination of substrate proteins. These two signaling pathways work together to modulate the function of the mitochondrial fusion pathway, thereby influencing the morphology and function of cellular mitochondria.^149^

Another study found that restricting dietary intake and activating AMPK extend life expectancy through peroxisome remodeling and mitochondrial network modulation.^150^ A restricted diet and activation of AMPK enable mitochondria to fuse, leading to large networks of mitochondria that enhance cellular energy production and biosynthetic capacity. Additionally, dietary restriction and AMPK activation promote peroxisome biogenesis and remodeling, enhancing cellular antioxidant and detoxification capabilities.^150^ These mechanisms may be important factors influencing dietary restriction and activation of AMPK in terms of extending lifespan.

Furthermore, studies conducted on *C. elegans* have shown that TORC1 signaling plays a vital role in promoting healthy aging, particularly in neurons. Furthermore, it has been observed that lifespan can be extended by modulating mitochondrial dynamics through the reduction of TORC1 signaling^151^ Specifically, the regulation of mitochondrial fusion and fission in neurons is controlled by TORC1 signaling. By inhibiting TORC1 signaling, mitochondrial fusion is promoted, which ultimately contributes to a longer lifespan.^151^ These findings provide important clues towards a better understanding of the mechanism behind the regulation of mitochondrial merging and how it influences organismal survival and lifespan extension.

In conclusion, aging has a negative effect on mitochondria, exhibiting reduced efficiency and alterations in mitochondrial dynamics. The effects of mitochondrial fission and fusion on longevity are dependent on the context and differ across various animal models. In *C. elegans*, mitochondrial fusion is controlled through the insulin/IGF-1 pathway and Cullin-RING ubiquitin ligase complex, and increased fusion promotes survival of older animals. AMPK activation and dietary restriction also modulate mitochondrial fusion as well as peroxisome remodeling, potentially contributing to their lifespan-extending effects. The underlying molecular mechanisms of this process require further investigation.

#### Ketogenic diet

The ketogenic diet (KD) is a therapeutic dietary approach that has been used clinically for several decades to manage symptoms of various diseases, such as epilepsy, autism spectrum disorder, and diabetes.^152–154^ The KD works by reducing carbohydrate intake and promoting FAO. The process of metabolic shift enables the production of ketones in the hepatocytes. These ketones are then utilized as a primary source of fuel for crucial organ systems such as the central nervous system, cardiomyocytes, and muscular tissue.^155^

According to research conducted on the BTBR^T+tf/j^ mouse, KD effect on mitochondrial dynamics is tissue-specific.^156^ In the brain, there are no difference between mitochondrial division and merging. In the liver, however, mitochondrial division and merging mediators are expressed at lower levels during the ketogenic diet. Specifically, expression levels of MFN2 and Drp1, closely related to merging and division respectively, are decreased while other proteins tend to decline. Therefore, it appears that the ketogenic diet alters the dynamics of mitochondria within the liver and reduces the expression of a number of regulators responsible for mitochondrial division and merging. The administration of ketogenic diets inhibites the fission of mitochondria and enhances mitochondrial activity in diabetic mice’s myocardium.^157^ It reduces mitochondrial number, increases mitochondrial size, improves respiratory rate, and increases heart ATP levels. In this study,^157^ the ketogenic diet affected mitochondrial dynamics through the regulation of AMPK/mTOR signaling. Particularly, the KD inhibites Drp1 expression and enhances MFN2 expression with the aim of reducing mitochondrial fragmentation and increasing mitochondrial fusion. Additionally, mitochondrial dysfunction is believed to be responsible for the occurrence of autism spectrum disorder. Dietary interventions, such as the ketogenic diet, may improve mitochondrial function.^158^

Overall, these findings indicate a significant association between KD and mitochondrial dynamics. It is, however, necessary to conduct further research in order to clarify the underlying mechanisms and develop appropriate strategies for disease treatment. With the development of research in this field, a deeper understanding of the correlation between function of the mitochondria and ketogenic diet can potentially pave the way for more effective therapeutic interventions in various diseases.

### Dysregulated mitochondrial dynamics in diseases

#### Genetic disorders

Mutations in genes regulating mitochondrial merging and division, including MFN2, Drp1, and Opa1, contribute significantly to the development of various neurological conditions. In particular, Charcot-Marie-Tooth disease (CMT) and autosomal dominant optic atrophy (ADOA) are genetic disorders associated with mitochondrial dysfunction.^159^

##### Charcot-Marie-Tooth disease (CMT)

is a commonly occurring form of inheritable neurological disorder, with autosomal dominant being the most common inheritance pattern, although autosomal recessive and X-linked subtypes also exist.^160^ Research efforts have focused on identifying disease-modifying therapies for the most frequently encountered genetic mutations, such as gap junction protein beta 1 (GJB1), peripheral myelin protein 22 (PMP22), myelin protein zero (MPZ), and MFN2.^160^ CMT manifests as the degeneration of the axons and myelin sheaths of peripheral nerves, leading to impaired nerve conduction velocity.^161^ Based on specific characteristics, CMT can be categorized into four major subtypes.^161^ CMT1 is characterized by demyelination and follows an autosomal dominant inheritance pattern. CMT2 is an axonal subtype and may be transmitted either *via* an autosomal dominant or a recessive inheritance pattern. CMTX is characterized by intermediate nerve conduction velocities and is typically associated with X-linked inheritance, although recessive and autosomal dominant intermediate variations are also known. The CMT4 subtype is demyelinating, but it shows an autosomal recessive inheritance pattern.

CMT2A is the most prevalent subtype among CMT2 patients, due almost exclusively to dominant mutations in the MFN2 gene that inhibit the fusion and motility of mitochondria.^162^ In a clinical setting, patients diagnosed with CMT2A frequently exhibit more severe symptoms and experience an earlier onset of the condition when compared to those with classic CMT.^163^ In addition, CMT2A manifests as peripheral neuropathy, progressive weakness of the muscles, impaired motor function, and may also affect the central nervous system (CNS), causing spinal cord or brain abnormalities. Nowadays, the treatment approach for CMT2A typically involves both general and specific treatments. For general treatments, coenzyme Q10 (CoQ10) supplementation may ameliorated the phenotype of CMT2A. CoQ10 is a vital component of the ETC in mitochondria, where it participates in energy production through ATP synthesis. Additionally, CoQ10 acts as an antioxidant, helping to combat harmful free radicals and defend cells against oxidative damage.^164^ Moreover, mitofusin agonists have emerged as a promising potential treatment for CMT2A, as they directly target the MFN2 mutations. Studies have demonstrated that mitofusin agonists can restore normal transport of mitochondria in the sciatic nerves of mice carrying the MFN2 Thr105Met mutation, suggesting a promising therapeutic approach for managing CMT2A.^165^

##### Autosomal Dominant Optic Atrophy (ADOA)

is a genetic disorder in which the neurons of the retina degenerate, developing atrophy of the optic nerve and impairment of vision.^166^ Study has demonstrated that ADOA is associated with mutations in three known loci (OPA4, OPA5, OPA8) and two genes (OPA1, OPA3) that encode proteins found in the inner mitochondrial membrane. In addition, X-linked or recessive optic atrophy can be attributed to other genes and loci (OPA2, OPA6, OPA7).^166^ At present, no cure has been found for ADOA, so the treatment is mainly supportive, focusing on physical therapy and the management of symptoms. It’s noteworthy that gene therapy offers potential therapeutic benefits for ADOA. A gene therapy aims to repair or replace the defective gene responsible for the disease, thereby restoring normal gene function. For example, a recent study has indicated that redirecting U1 snRNA to non-canonical splice sites can effectively correct splicing defects in the OPA1 gene. This, in turn, can enhance the production of properly spliced transcripts of OPA1, potentially overcoming haploinsufficiency.^167^

#### Neuro-degenerative diseases

##### Parkinson’s Disease (PD)

is a neurodegenerative disease due to cell death or degeneration in the region of the brain known as the substantia nigra, leading to the deterioration of dopamine neurons. Dopamine neurons are crucial for controlling body movement and coordination; hence PD patients typically exhibit symptoms such as muscle rigidity, tremors, and bradykinesia.^168^ Over the past few decades, accumulated evidences point towards a connection between PD and mitochondrial dysfunction. Mild deficiency in Complex I activity and oxidative damage have been observed to contribute to the development of neurodegeneration in PD.^169^ Changes in antioxidant levels and targets of oxidation have also been observed in PD, further supporting the oxidative stress’ involvement in the disease. Recent studies have identified various genetic changes in the genes responsible for encoding proteins that are targeted to mitochondria. Additionally, gene variants related to mitochondrial dynamics and function, such as DJ-1, PINK-1, Parkin, and leucine rich repeat kinase 2 (LRRK2), have been implicated in the association between mitochondria and PD.^170^

An essential characteristic of mitochondrial dynamics is the distribution of mitochondria to synapses, supporting synaptic function, and ensuring the health of mitochondria in general. The disruption of mitochondrial dynamics might represent the initial event in the neurodegenerative process of PD.^171,172^ The intricate and essential function of mitochondrial fusion/fission machinery in the formation of synapse and dendritic spine formation in neurons has been well established. Inhibiting mitochondrial fragmentation results in diminished mitochondrial concentration within dendritic spines and a decrease in synaptic development, while stimulating fission promotes the formation of synapses.^173^ For instance, mitochondrial dynamics contribute to the development of PD based on findings from studies using toxin-induced PD models. Moreover, absence of Drp1 hampers the distribution of mitochondria to synapses and disrupts synaptic function.^174^

Mutations in certain genes linked to familial PD have also highlighted the important role that mitochondrial dynamics play in this disorder. Emerging evidence strongly indicates that gene mutations influencing mitochondrial dynamics are crucial to the development of PD.^175^ Parkin, a protein encoded by the PARK2 gene, is implicated in PD.^176^ A cytosolic ubiquitin E3 ligase protein, Parkin plays an important role in the process of ubiquitin-dependent proteolysis and is known to affect mitochondrial health through its regulatory role in mitochondrial dynamics.^177^ In drosophila model, defective mutation of Parkin results in enhanced oxidative stress sensitivity, loss of dopaminergic cells, and severe mitochondrial dysfunction characterized by swollen mitochondria and fragmented cristae.^178^ Similarly, mutations in other PD-associated genes, such as PINK1, which codes for a mitochondrially-targeted kinase, add further evidence to a correlation between mitochondrial dynamics and PD pathogenesis.^176^

##### Alzheimer’s disease (AD)

accounts for a significant percentage of impaired cognitive function in elderly people. The main pathological features in AD are degeneration of brain neurons and synapses, as well as excessive deposition of neuronal proteins, especially β-amyloid (Aβ) plaques and tau protein tangles.^179^ Mitochondria contribute significantly to the development of AD. Metabolic disturbances in AD are well-documented, with reduced brain metabolism being a prominent feature.^180^ Deficiencies in key enzymes of OXPHOS in the brain, alongside damage to mitochondria and increased ROS are consistently documented in AD.^181^ Alterations in calcium homeostasis have also been observed among AD patients, which suggest that mitochondrial dysfunction may contribute to dysregulation of neuronal calcium levels in AD.^182^ Furthermore, genetic markers in mtDNA are associated with increased risks of AD.^183^

Usually, neurons of AD patient display both malfunctioning mitochondria and unfavorable mitochondrial dysfunction. For instance, compared to a control group of age-matched neurons, neurons from AD show a marked reduction in the proportion of normal mitochondria and a notable rise in the proportion of mitochondria with broken cristae.^184^ In addition, Opa1, Drp1, MFN1/2 expression are significantly lower in AD, whereas Fis1 levels are higher in AD.^185^ Mitochondrial dynamics impact the pathological changes of AD involved in several signaling pathways, including Ca^2+^, AMPK, and nitric oxide signaling pathways. Non-canonical Wnt-5a/Ca^2+^ signaling is crucial in mitochondrial dynamics, and its activation shields hippocampal neurons from Aβ oligomer-induced damage.^186^ AdipoRon, an adiponectin receptor agonist that significantly improves synaptic efficiency, enhances fusion of mitochondria, and mitigates tau hyperphosphorylation in SY5Y cells, rescuing memory deficits in P301S tau transgenic mice.^187^ Mechanistically, AMPK/GSK3β and AMPK/SIRT3 signaling participate in enhancing the positive effects of AdipoRon on mitochondrial dynamics along with tau accumulation.^187^ Besides, Aβ-induced nitric oxide mediates mitochondrial fission and neuronal injury through Drp1 S-nitrosylation, which may facilitate the progression of AD.^188^ Therefore, prevention of Drp1 S-nitrosylation eliminate these neurotoxic events.^188^

##### Huntington’s disease (HD)

is a progressive neurodegenerative condition that is both fatal and progressive, with limb tremors and decreased cognition, and it is inherited autosomally dominantly.^189^ The brains of HD patients were subjected to histopathological examination, which revealed that multiple brain regions such as the caudate, putamen, and cortex of the striatum as well as the subthalamus and hypothalamus had been affected. HD-associated mutations are caused by a gene that contains an enlarged repeat of the polyglutamine encoding sequence (CAG repeat), which is located within exon 1 in the HD gene.^190^

Mechanisms responsible for the degeneration of neurons in individuals with HD are not yet fully comprehended. Current research efforts to understand the pathogenesis of HD have primarily focused on investigating abnormalities in mitochondrial dynamics, especially increased mitochondrial division, resulting in malfunctioning mitochondria, and deficits in the trafficking of axons and synaptic transmission in neurons suffering from HD.^191–194^ Several genes that are implicated in the ETC and mitochondrial structure, among which are Fis1, Drp1, MFN1/2, Tomm40, Opa1, and CypD, were examined in individuals suffering from stage III and IV HD. It was found that Fis1 and Drp1 expression increased in HD patients, while MFN1/2, Tomm40 and Opa1 expression decreased. These abnormalities impact mitochondrial function, neuronal transport, and cell death, potentially accelerating the progression of HD.^191^ Another study found that increased mutant huntingtin (HTT)-Drp1 interaction alters Drp1 structural and functional properties, causing increased mitochondrial division and decreased ATP production, resulting in neuronal dysfunction.^193^

#### Metabolic diseases

##### Diabetes

refers to a metabolic disease associated with insulin resistance, inadequate insulin secretion, and abnormal glucose metabolism. The etiology and progression of this disease are associated with several factors. Evidences suggest that mitochondrial fragmentation is responsible for insulin resistance. Mitochondrial dynamics disturbances, particularly mitochondrial fission, may exacerbate insulin resistance and impaired glucose metabolism of hybrid cells bearing mitochondrial haplogroup B4, thereby facilitating the onset and progression of diabetes.^195^

Evidence demonstrated that palmitate (PA) overabundance resulted in the fragmentation of mitochondria and increased levels of the mitochondrial proteins Fis1 and Drp1 in maturing C2C12 muscle cells.^148^ This fragmentation correlates with elevated levels of oxygen-free radicals, depolarization of mitochondria, decreased ATP synthesis, as well as impaired glucose uptake upon insulin stimulation. Furthermore, inhibition of Drp1 using genetic and pharmacological approaches effectively mitigate C2C12 cells’ mitochondrial depolarization, fragmentation, and insulin resistance as a result of PA-induced mitochondrial fragmentation, suggesting a potential therapeutic strategy for managing these detrimental effects.^148^ Besides, hyperglycemia during gestational diabetes mellitus poses a threat to the functioning of placental tissue. It disrupts mitochondrial fusion thereby alters the equilibrium of the dynamics of mitochondria in placental tissue. When mitochondrial fission is chemically inhibited in cultured placental trophoblast cells, the mitochondrial fusion is alternatively activated.^196^ Inhibiting mitochondrial fission results in a reduction in the generation of ROS, expression of markers for unfolded proteins in mitochondria, and mitochondrial depolarization. Additionally, it improves insulin sensitivity in placental cells during hyperglycemia.^196^

Diabetes development is closely associated with impaired function of pancreatic β-cells, which can be triggered by various factors, including ER stress.^197^ The expression of Drp-1 significantly enhance apoptosis induced by ER stress in the Drp1 WT activated β-cell line, as opposed to Drp1 K38A (a dominant negative mutant of Drp1) inducible β-cell line.^198^ Rhein, a compound derived from rhubarb and belonging to the anthraquinone family, displays potential in ameliorating glucose metabolism disorders in mice with diabetes. Mechanically, rhein prevents the apoptosis of pancreatic beta-cells caused by increased glucose levels *via* stabilizing mitochondrial morphology. Through its localization at β-cell mitochondria, rhein can preserve mitochondrial integrity through inhibition of mitochondrial fission protein Drp1, which is induced by hyperglycemia.^199^

In summary, diabetic individuals often display resistance to insulin and abnormal glucose metabolism. Recent studies have suggested that mitochondrial fission, which affects mitochondrial dynamics, can contribute to insulin resistance, pancreatic beta-cell malfunction, and the production of reactive oxygen species. Therefore, inhibiting mitochondrial division may have therapeutic potential for managing diabetes.

##### Non-alcoholic fatty liver disease (NAFLD)

comprises a range of hepatic diseases manifested by an abnormal fat deposition within the liver in the absence of an excessive alcohol consumption history. Steatosis, which is the accumulation of fat in >5% of hepatocytes, is a hallmark of NAFLD.^200^ Diabetes, insulin resistance, metabolic syndrome, and mutations in the genes PNPLA3 (patatin-like phospholipase domain-containing protein 3) and TM6SF2 (transmembrane 6 superfamily member 2) are contributing factors to NAFLD.^201–203^ Accumulating evidence suggests that structural and bioenergetic changes to the mitochondria are involved in the pathogenesis causing NAFLD, which may progress into non-alcoholic steatohepatitis.

The occurrence and advancement of NAFLD are closely linked to mitochondrial fission. According to in vitro studies, treating hepatocytes with PA results in mitochondrial fragmentation, impairing transmembrane voltage, excretion of Cyt C, and increased ROS activity.^204^ Using high-fat diet induced NAFLD as a model, increased protein levels of Drp1, mitochondrial fragmentation, as well as heightened hepatocyte lipolysis are observed in liver.^205^ Moreover, inhibition of mitochondrial division through expressing the dominant-negative fission mutant (Drp1-K38A) alleviates the oxidative stress and impairment of liver function resulting from excess intake of fat, exerting protective effect against liver steatosis.^205^ This study offers mechanistic evidence supporting the role of mitochondrial fission in regulating liver lipid metabolism and oxidative injury, both of which are linked to the development of NAFLD.

On the other hand, hepatocytes from mice with NAFLD exhibit lower expression levels of MFN1, which correlates with the development of steatohepatitis.^206,207^ Additionally, treatment of hepatocytes with PA leads to downregulation of both transcript and protein levels of MFN2.^208^ Furthermore, diminished levels of MFN2 are observed in livers of patients with non-alcoholic steatohepatitis (NASH) as well as in murine models of NAFLD. Interestingly, deletion of hepatic MFN2 significantly promotes inflammatory responses, accumulation of triglycerides, fibrosis, and HCC in mouse models of NASH, whereas restoring MFN2 expression using adenovirus in mutant (liver-specific) mice lacking MFN2 observably alleviate disease symptoms of NASH.^209^

The underlying mechanism through which fission in mitochondria facilitates the development of NAFLD remains poorly understood. Due to mitochondria’s central role in coordinating hepatic metabolism of lipids, studies have proposed that division of mitochondria is connected with ROS and dysfunction within the mitochondria, which potentially contribute to NAFLD. Indeed, after exposing to PA, HepG2 cells display more mitochondrial fragmentation, elevation of superoxide levels, and overall oxidative stress.^208^ Importantly, mitochondrial fragmentation occurs prior to the generation of ROS, with signs of fragmentation observed as early as 12 hours, whereas increased ROS generation is not evident until after 12 hours of exposure to PA.^208^ As previously reported, overexpressing Drp1-K38A (the dominant-negative fission mutant) reduces oxidative stress and ROS levels in the context of hyperglycemia.^210^ Furthermore, Drp1-K38A expression lowers oxidative damage and inflammation within a model of NAFLD.^205^ Suppression of mitochondrial fission causes proton leak when PA is present in vitro. Given that proton leak refers to the nonproductive dissipation of energy during OXPHOS, the increased proton leak contributes to reduction of ROS under the conditions of inhibiting of mitochondrial fission.^205^

#### Cardiovascular diseases

##### Ischemia-reperfusion injury

Acute myocardial infarction accounts for a significant amount of disability and mortality around the world. In order to limit the size of myocardial infarction and reduce acute myocardial ischemic injury, rapid and effective reperfusion of the myocardium is preferred for patients with myocardial infarction. This can be achieved through either primary percutaneous coronary intervention or thrombolysis. However, it is important to note that reperfusion itself can lead to cardiomyocyte death, which is known as myocardial reperfusion injury.^211^ Currently, no effective treatments are available for this condition.^212^ Ischemia-reperfusion injury (IRI) to the heart entails a complicated process resulting in tissue damage and cells dying. Ischemia causes a lack of oxygen and nutrients in the heart, which results in anaerobic metabolism and the production of lactic acid. When blood flow is reintroduced during reperfusion, it can trigger harmful events such as the ROS production, calcium overload, inflammation, mitochondrial dysfunction, and ER stress. These events can lead to cellular dysfunction and ultimately cell death.^212,213^ Therefore, comprehending the intricacies of cardiac IRI is essential for developing effective preventative measures or minimize the damage caused by this phenomenon.

Increasing evidences point to the role played by mitochondrial dynamics in IRI. Ischemia cause mitochondrial fragmentation, which mainly depends on Drp1 and links to increased release of ROS and calcium overload.^10,11^ High level of ROS can induce mitochondrial fission during acute IRI, while pre-treatment with scavengers of mitochondrial ROS-SkQ1 or Trolox, reduces the phosphorylation of Ser616 in Drp1 and mitochondrial fission after IRI.^43,214^ During acute IRI, cytosolic calcium overload can activate calcineurin, which is responsible for activating Drp1 by dephosphorylating Ser637, inducing Drp1-mediated mitochondrial fission.^215^

Increasing mitochondrial fusion or inhibiting mitochondrial fission can provide a protective effect on heart against IRI. Transfection of these cells with mitochondrial fusion proteins, including MFN1/2 or Drp1K38A (a recessive variation in Drp1), resulted in more cells with prolonged mitochondria, reduce mitochondrial permeability transition pore sensitivity, along with decreased cellular death following IRI.^10^ Besides, in the prediabetic rats, administration of Mdivi-1 at any time point during IRI effectively reduces ROS production, mitochondrial swelling and depolarization, as well as dynamic imbalance.^216^ Additionally, dual-specificity protein phosphatase1 (DUSP1) is involved in regulating cardiac metabolism, and its expression decreases after acute cardiac IRI. Nevertheless, the reintroduction of DUSP1 suppresses Mff activation, thus mitigating fatal mitochondrial fission by deactivating the JNK pathway.^217^ Moreover, in the context of IRI, there is an upregulation of mitochondrial calcium uniporter (MCU), which triggers calpain activation. Calpain activation, subsequently, triggers the downregulation of Opa1, ultimately leading to mitochondrial fission.^218^ MCU suppression using Ru360 during IRI appears to minimize the area of myocardial infarction and apoptosis in cardiomyocytes, reduce mitochondrial fractures and promote mitochondrial fusion and mitophagy.^218^ Furthermore, Opa1 inactivation and mitochondrial fusion is a significant phenomenon during IRI, but it is reversible when treated with melatonin.^219^ Melatonin helps to normalize the fusion of mitochondria associated with Opa1 *via* AMPK pathway, which corrects excessive mitochondrial division, promotes mitochondrial energy metabolism, maintains mitochondrial function, and blocks mitochondrial apoptosis in cardiac myocytes.^219^

##### Heart Failure (HF)

refers to a condition in which the heart cannot provide enough oxygen and nutrients to the body, leading to decreased physical function and often accompanied with symptoms such as breathing difficulty, fatigue, and edema.^220^ An impaired mitochondrial function has been identified as a key characteristic of HF, which decreases ATP synthesis and increases ROS generation, leading to a reduced energy supply to the heart muscle cells, impairing contractile function and worsening HF progression.^221^ Increasing studies indicate that improper mitochondrial merging and division augment the development of HF. Reduced Opa1 expression and small mitochondrial fragmentation appear in the failing hearts, indicating decreased mitochondria fusion in the failing hearts.^222^ In mice, specific deletion of Yme1l in the heart activates OMA1, leads to increased Opa1 degradation, fragmented mitochondria, and altered metabolism of the heart, finally, resulting in dilated cardiomyopathy and HF.^223^ However, deletion of Oma1 prevents cleavage of Opa1, which in turn rescues cardiac function and mitochondrial morphology. It is noteworthy that high-fat feeding of mice or deletion of the Yme1l gene in skeletal muscle restores heart metabolism and preserves cardiac activity, irrespective of inhibiting mitochondrial fission.^223^

Promotion of mitochondrial fusion prevents the aggravation of HF. Omentin1, a newly identified adipokine, has been shown to safeguard against HF induced by myocardial ischemia.^224^ In mice with HF, administering omentin1 enhances mitochondrial fusion while decreasing mitochondrial fission, with an upregulation of Opa1 and MFN2, whereas a decrease in Drp1(Ser616).^224^ Doxycycline (DOX) exerts protective effects in animal models of HF. Mechanically, in H9c2 cardiomyocytes, the action of DOX alleviating the severity of HF involves reducing mitochondrial depolarization and fragmentation induced by oxidative stress, as well as positively modifying the levels of Opa1, MFN2, and Drp1.^225^

##### Cardiomyopathy

Maintaining a balance between mitochondrial division and merging is crucial for maintaining the structural integrity of the myocardium. Research has shown that a disturbance in mitochondrial fusion in mature cardiac tissue can lead to cardiomyopathy and disrupt cardiac homeostasis in mice.^226^ In *Drosophila*, knocking down of mitochondrial assembly regulatory factor (MARF), which is similar to Opa1 or MFN1/2, causes mitochondrial fragmentation and dilated cardiomyopathy.^227^ Structure and function abnormalities of the heart, causing diabetes-related cardiomyopathy (DCM), are commonly associated with diabetes.^228^ Specifically, for *db/db* mice, diabetes-induced hearts exhibite increased mitochondrial fission accompanied by a significantly lower expression of MFN2. However, reintroducing MFN2 into hearts suffering from diabetes preventes the progression of DCM by inhibiting mitochondrial fission.^229^

Paeonol, a bioactive compound with potential pharmacological benefits in protecting the heart and mitochondria, promotes the fusion of mitochondrial mediated by Opa1, alleviates the accumulation of oxidative stress in mitochondria, sustains mitochondrial respiration, and boosts cardiac performance both in vitro and in vivo models involving DCM.^230^ Paeonol has been found to promote fusion of mitochondria through Opa1 activation by activating STAT3. This mechanism involves the transcription factor attaching to Opa1 promoters and subsequently increasing its transcription.^230^ The deficiency of the Mff gene in mice leads to dilated cardiomyopathy, which ultimately results in heart failure and death. Mutant tissue exhibits lower mitochondrial content and reduced activity of the respiratory chain, with an increase in mitophagy. However, the removal of MFN1 simultaneously restores respiratory chain function, life span, and heart dysfunction, thereby preventing Mff-deficient cardiomyopathy.^231^

#### Cancer

**Fig. 5:**
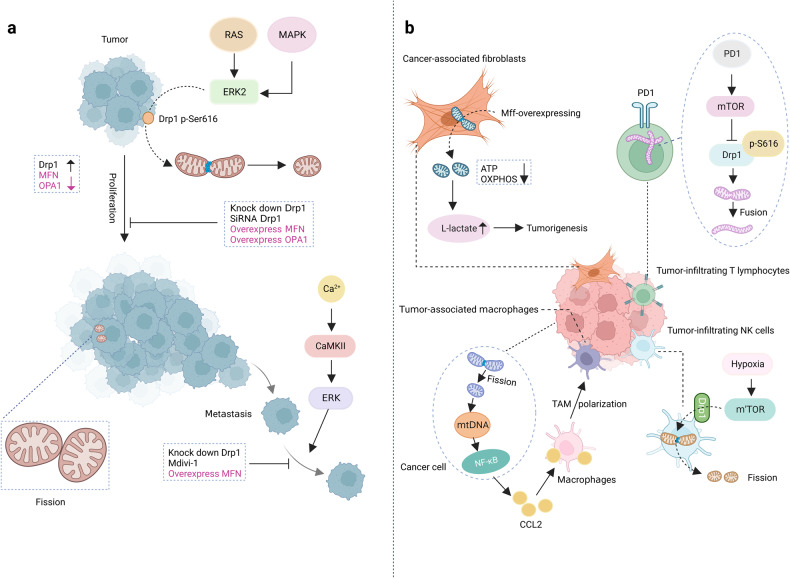
Mitochondrial dynamics in cancer. **a** Mitochondrial division in cancer cells show increased levels of Drp1 and decreased levels of MFN2. Cancer-promoting factors such as the oncogene Ras or activation of the MAPK pathway can trigger the phosphorylation of Drp1 at Serine 616 by ERK2, leading to mitochondrial fission and increased fragmentation. **b** Mitochondrial fission induces glycolytic reprogramming in CAFs, driving stromal lactate production, and tumor growth. TILs commonly experience exhaustion during cancer progression. PD-1 signaling inhibits mitochondrial fragmentation in T cells by downregulating Drp1 phosphorylation on Ser616, likely through regulation of the ERK1/2 and mTOR pathways; TAMs recruitment and polarization is facilitated by mitochondrial fission causing cytosolic mtDNA stress, which increases CCL2 secretion by cancer cells; tumor-infiltrating NK cells have small and fragmented mitochondria due to excessive fission caused by the sustained activation of mechanistic target of mTOR-Drp1 in the hypoxic TME. This mitochondrial fragmentation leads to decreased cytotoxicity and enables tumors to evade NK cell-mediated surveillance. CAFs cancer-associated fibroblasts, TIL tumor-infiltrating T lymphocytes, TAMs tumor-associated macrophages

Recent studies provide mounting evidence that mitochondria play a crucial role in tumorigenesis and progression. In addition to their essential bioenergetic functions, mitochondria are also responsible for cancer anabolism, calcium homeostasis, redox regulation, gene transcription, and the orchestration of cell fate.^232^ Moreover, numerous immune functions depend on the proper efficiency of mitochondrial metabolism in immunocytes.^233^ Dysregulations of mitochondrial dynamics are implicated leading to the occurrence and development of various cancers, influencing aspects such as tumor cell proliferation, metastasis, drug resistance and tumor microenvironment (TME) (Fig. 5), indicating that addressing mitochondrial dynamics may be a promising anticancer therapeutic approach.

##### Cell proliferation

The uncontrolled growth of cells, disrupted cell cycle regulation, in addition to abnormalities in programmed cell death, are hallmarks of cancer.^234^ A significant role is played by mitochondrial dynamics during these processes. Therefore, recent research has provided compelling evidence linking the merging and division of mitochondria to the advancement of different types of cancer. Mitochondrial division is detected in cancerous cells,^235^ and hindering this process causes reduced proliferative activity and enhanced death of cells in some cancer model systems. For instance, lung cancer samples obtained from patients show the same pattern of increased Drp1 and decreased MFN2 levels compared with tissue adjacent to healthy lung tissue. Manipulating the mitochondrial network formation, such as through overexpression of MFN2 or inhibition of Drp1, has been found to decrease growth and increase the occurrence of spontaneous apoptosis in lung cancer cells.^236^ Similarly, knocking down Drp1 stimulates increased numbers of mitochondrial elongation, proliferation retardation and an increase in apoptosis of both HCT116 and SW480 human colon cancer cells.^236^

Furthermore, compared to normal gastric mucosal tissue, MFN2 expression on gastric cancer cells is downregulated, its level negatively correlates to tumor growth, suggesting a potential anti-tumor function for MFN2.^237^ A higher level of MFN2 appears to restrict gastric cancer cell reproduction in vitro.^238^ The expression of FUN14 domain-containing 2 (FUNDC2) is increased in HCC at the transcriptional level. Notably, elevated levels of FUNDC2 expression correlate with reduced patient survival, while its knockdown has been shown to inhibit liver tumor development in mice. The mechanism of action involves FUNDC2’s amino terminus interacting with the GTPase domain of MFN1, which inhibits MFN1 activity that normally promotes the fusion of the OMM.^237^

Dysfunctional mitochondrial dynamics can influence the signaling of intracellular carcinogens. The activation of ERK2 by oncogene Ras or the MAPK pathway can trigger the phosphorylation of Drp1 at Ser616, leading to mitochondrial fragmentation. This process has been linked to tumor growth.^239^ Moreover, reducing the expression of Drp1 dampens the growth of tumors caused by MAPK-mediated malignancies.^239^ In HCC, the extracellular matrix-associated protein CCBE1 significantly promote mitochondrial fusion and inhibit the progression of HCC. The function of CCBE1 involves inhibiting fission of mitochondria by impeding the localization of Drp1 to mitochondria by preventing phosphorylation of Drp1 at Ser616.^240^ In cervical carcinoma Hela cells, MFN2 inhibits proliferation and cell-cycle by inhibiting the expression of key proteins, including NF-κB p65, Myc, and mTOR, and by suppressing Ras protein activity.^241^

##### Metastasis

Tumor metastasis is closely linked with mitochondrial fission. Metastatic breast cancer cells exhibit an increase in mitochondrial fragmentation as a result of their elevated levels of active Drp1 and reduced MFN1 expression.^85^ Mitochondria elongation or clustering can greatly reduce the metastasis potential in cancerous breast cells, which can be caused by either Drp1 deficiency or MFN1 overexpression. Alternatively, silencing the MFN1 gene results in the fragmentation of mitochondria in breast cancer cells, which in turn increases their ability to metastasize.^85^ Rab32, a protein related to Ras, is significantly upregulated in glioblastoma multiforme (GBM), particularly in its highly malignant mesenchymal form. Reduction of Rab32 levels decreases the migration and invasion potential in GBM cells.^242^ Mechanisms underlying Rab32 promoting GBM aggressiveness involves the ERK/Drp1 pathway, i.e., Rab32 facilitates the transport of Drp1 into mitochondria, where it recruits ERK1/2 to phosphorylate the Ser616 region of Drp1.^242^

An elevated Drp1 expression has been connected to malignant thyroid tumors. By genetically and pharmacologically blocking Drp1 activity, it was possible to attenuate the migration ability of thyroid cancer cells.^243^ In addition, excessive mitochondrial fission is observed in highly metastatic HCC. One of the main downregulated candidates, MFN1, is closely related to the metastasis of HCC.^244^ The protein MFN1 has been found to inhibit the growth, spread, and movement of HCC cells in both living organisms and laboratory settings. This is achieved through its ability to encourage the merging of mitochondria. Conversely, when MFN1 is absent, the dynamics of mitochondria are disrupted, leading to the process of EMT in liver cancer cells.^244^

The activation of the Ca^2+^/CaMKII/ERK/FAK pathway has been shown to be important in the promotion of focal-adhesion dynamics and lamellipodia formation during HCC cell reprogramming, which is largely facilitated by mitochondrial fission.^245^ A high level of EGFR expression is present within mitochondria from highly aggressive non-small cell lung cancer (NSCLC) cells, which can stimulate NSCLC invasion and metastasis by interacting with MFN1 and causing disruption in mitochondrial fusion.^246^ Furthermore, the protein SIRT4 achieves cancer cell invasion and expansion *via* impeding mitochondrial dynamics and inhibiting the ERK-Drp1 pathway.^247^

Overall, proteins that promote mitochondrial fission tend to be elevated in tumorous and metastasized patient samples in comparison with the normal tissue. The increased levels of such proteins are commonly associated with unfavorable clinical outcomes and affect numerous cellular characteristics critical to the progression of tumors, such as tumor growth, migration, and invasion. These observations underscore the usefulness of the mitochondrial division mechanism for therapeutic purposes in treating metastatic disease.

##### Drug resistance

Mitochondrial dynamics have been identified as crucial for drug resistance development in cancerous cells.^248^ Breast cancer stem cells (BCSCs) play a crucial role in chemotherapy resistance and recurrence in breast cancer. Studies have shown that compared to 2D cultured parent cells, BCSCs exhibit significantly increased levels of Fis1 and MFN1 proteins. Treatment with AZD5363 (Capivasertib) is believed to affect mitochondrial dynamics in BCSCs by suppressing MFN1 expression, thus increasing the sensitivity of BCSCs to doxorubicin chemotherapy.^249^ Furthermore, Transferred breast carcinoma cells have been found to spread to organs that display a more favorable microenvironment characterized by elevated mitochondrial division mediated by Drp1 and mitochondrial elongation factor (MIEF)1/2. The pharmacological inhibition of Drp1 has been shown to restore sensitivity to cisplatin (DDP) and restrain diffuse neoplastic cell awakening. These findings suggest that modulating mitochondrial dynamics may be a potential strategy to prevent metastatic chemotherapy resistance.^250^ Additionally, Opa1 contributes to resistance to gefitinib, an inhibitor of tyrosine kinases, in a lung adenocarcinoma (LUAD) cell line.^251^ Tht gefitinib-resistant LUAD cells exhibit elongated mitochondria with narrower cristae and elevated Opa1 expression levels. Inhibiting Opa1, either through genetic or pharmacological means, restores mitochondrial morphology and sensitizes the cells to the discharge of Cyt C accompanied by death of the cells induced by gefitinib.^251^

DDP is widely utilized in advanced cancer therapy. The Mff protein is extensively expressed in HCC cell lines and tissues resistant to DDP.^252^ In Huh-7/DDP cells, knocking down Mff prevents mitochondrial division and reduces Drp1 levels, which enhances the sensitivity of Huh-7/DDP xenografts to the treatment with DDP in vivo.^252^ Meanwhile, another study also revealed that Mdivi-1 sensitizes chemo-resistant breast and lung cancer cells to DDP.^253^ However, in ovarian carcinoma, decreased fission protein expression was found to enhance cisplatin resistance.^254^ In DPP-resistant SKOV3 cells, a markedly increased mitochondrial length, down-regulated Drp1 expression and up-regulated MFN2 expression are observed. Furthermore, inhibiting Drp1 or increasing MFN2 expression can enhance SKOV3 cells’ resistance to DDP.^254^ MFN2 expression increases significantly in leukemic Jurkat cells that are resistant to doxorubicin treatment. Additionally, there is a notable increase in the expression of the OXPHOS complex (respiratory system) and the synthetase of ATP in these cells. When MFN2 is knocked down using CRISPR, the LD50 of doxorubicin is lower than in wild-type cells exposed to the drug, indicating that mitochondrial fusion hinders the cell’s ability to respond to chemotherapy.^255^

An effect of bone marrow-derived MSCs on T-cell acute lymphoblastic leukemia (T-ALL) cells has been found to promote chemoresistance by altering the mitochondrial dynamics. When T-ALL cells are cultured with MSCs, their mitochondrial structure undergoes a shift from elongation to fragmentation. This shift is due to the phosphorylation of Drp1 at residue Ser616, which is primarily mediated by the activation of extracellular signal-regulated protein kinase.^256^ Chemoresistant cells can induce high-mobility group box 1 (HMGB1) to be released into conditioned media following the death of cells, which triggers the phosphorylation of Drp1 through the receptor for advanced glycation end product (RAGE). Based on these results, it appears that HMGB1 released by dead tumor cells promotes chemical resistance as well as tumor development through RAGE-dependent ERK/Drp1 phosphorylation.^257^

In summary, mitochondrial dynamics play a crucial role in the development of drug resistance in cancer cells. Targeting specific molecules that are associated with the merging or division of mitochondria has been shown to increase the effectiveness of chemotherapy and targeted therapy in inhibiting tumor growth. Therefore, it is crucial to understand the mechanisms that underlie drug resistance and to develop efficient methods to combat it, in order to improve cancer treatment outcomes.

##### Tumor microenvironment.

Tumor immunotherapy has achieved several advancements in recent years that have resulted in increased survival rates for cancer patients.^258^ However, the efficacy of immunotherapy is limited as only a portion of patients exhibit a response to the treatment. This is partly due to the hindrance of immune cell migration and infiltration caused by TME, which leads to the exhaustion of immune cells.^259^ Recent research suggests that mitochondrial dynamics have a profound effect upon immune surveillance within the TME.

During HCC progress, the interaction between TME and HCC cells is of vital importance. The dynamic modifications in mitochondrial division and merging are pivotal in maintaining mitochondrial homeostasis and mtDNA distribution.^260^ Fission mediated by Drp1 induces the cytoplasmic stress of mtDNA, which promotes the release of CCL2 by HCC cells *via* activating the TLR9-mediated NF-B pathway, thereby facilitating M2-polarization and the recruitment of tumor-associated macrophages (TAMs).^261^ Moreover, blocking CCL2/CCR2 signaling by antagonists significantly reduces TAM invasion thereby restrains the advancement of HCC in orthotopic murine models. These observations suggest that mtDNA damage induced by mitochondrial fragmentation enhances invasion of TAMs and HCC progress *via* CCL2 secretion.^261^

Tumor-infiltrating T lymphocytes (TIL) commonly display exhaustion, and their fate is believed to be regulated by mitochondrial dynamics.^81^ In different types of TME, various signaling molecules impact mitochondrial dynamics in distinct ways, ultimately affecting T cell function. Specifically, programmed cell death-1 (PD-1) signaling is found to downregulate the T-cell response through Drp1-dependent mitochondrial fission.^262^ The activation of T-cells leads to mitochondrial fragmentation, but this process is inhibited by PD-1 signaling. This inhibition occurs through a reduction in the phosphorylation of Drp1 at Ser616, which is likely regulated by the mTOR and ERK1/2 pathways. In an MC38-derived murine tumor mass, CD8^+^ T cells expressing PD-1 have reduced Drp1 activities and longer mitochondria compared to their PD-1-negative counterparts, which may explain their reduced motility and proliferation.^262^ In hypoxic nasopharyngeal carcinoma microenvironments, mitochondrial fusion protein MFN1/2 expression is decreased, leading to small and fragmented mitochondria in TILs.^263^ It is affected due to increased expression of exosomal miR-24 in response to hypoxia, which inhibits its target gene Myc. Myc directly regulates the transcription of MFN1, which influences mitochondrial morphology, thereby establishing a miR-24-Myc-MFN1 axis. Consequently, increased miR-24 level results in decreased MFN1 expression and small and fragmented mitochondria. However, removing exosomal miR-24 can reverse T cell depletion and reduce tumor cell growth.^263^ Thus, mitochondrial dynamics and metabolites are essential for optimizing T cells’ anti-tumor function.

TAMs make up the largest component of immunological cellular populations in TME. TAMs have been classified for alternately active (M2) macrophages within the TME, where they facilitate metastasis, angiogenesis, and suppression of the immune in a variety of cancers.^264^ Compared to normal macrophages, the mitochondrial morphology of TAMs is more static and unstable.^56^ They exhibit more elongated or sheet-like structures, while normal macrophages have more circular structures. In addition, TAMs have a lower mitochondrial membrane potential, leading to reduced ATP synthesis.^56^ Inhibiting the expression of the FAM73b protein promotes mitochondrial fission and increases IL-12 production. This transition in TAMs leads to activation of T cells enhancing antitumor immunity.^56^

Tumor-infiltrating NK cells take part in immunological reactions against cancerous cells through destroying them and secreting cytokines. Nevertheless, within immune-suppressive TME, inhibitory proteins generated from malignant cells leads to an abnormal function of NK cells, resulting in the escape of tumor cells. In liver cancers of humans, NK cells that infiltrate tumors display small and shattered mitochondria. The fragmentation of mitochondria is referred to aberrant mitochondrial metabolism, decreased cytotoxicity, enabling tumor evasion of NK cell detection.^265^ Oxygen shortage in TME is responsible for the sustained activity of mechanistic targets of the mTOR-Drp1 pathway in NK cells, which leads to excessive fission of mitochondria. However, inhibiting fragmentation of mitochondria improves mitochondrial ATP synthesis and the tumor-fighting ability of NK cells.^265^ In triple negative breast cancer (TNBCs), ELK3 (E26 transformation-specific transcription factor ELK3)-dependent mitochondrial fission/fusion status is a main determinant of NK cell-mediated immune responses. ELK3 expression is inversely correlated with Mid51, a protein involved in mitochondrial dynamics. This connection between Mid51 and ELK3 has a significant impact on mitochondrial dynamics, which in turn affects the anti-tumor effectiveness of NK cells for treating TNBCs.^266^

Cancer-associated fibroblasts (CAFs) are extremely plastic cells in TME, are closely associated with tumorigenesis and progression. Multiple studies have shown that CAFs participate in metabolic reprogramming of tumor and exert regulatory effects *via* their dysregulation of metabolic pathways. More specifically, oxidative stress induced by tumor cell drives an initiation of mitophagy, autophagy, and glycolysis in CAFs.^267,268^ Metabolic reprogramming towards glycolytic metabolism of fibroblasts occurs as a result of the overexpressing of Mff, which triggers extensive mitochondrial fragmentation and mitochondrial dysfunction. Furthermore, Mff-overexpressing fibroblasts display that they are depleted of ATP and secrete L-lactate, leading to accelerated early tumor growth.^269^ These results suggest that mitochondrial division leads to a reprogramming of glycolysis in CAFs, contributing to the production of lactate in the stroma, as well as the early development of tumors.

The regulatory T cell (Treg), a CD4+ T cell subset that exhibits immunosuppressive properties, has a crucial function in restricting the activity of T lymphocytes inside the TME and promotes the development of tumors. Tregs undergo metabolic reprogramming that permits utilization of alternative substrates and engagement of various metabolism pathways for fulfilling the energy requirements in TME.^270^ Tregs often switch their metabolism towards mitochondrial OXPHOS from glycolysis, which is partly regulated by Foxp3 transcriptional activity. Moreover, the expansion of TIL-Tregs and their ability to suppress the immune system rely heavily on mitochondrial metabolic activity.^271^ For example, the capacity of Tregs in suppressing cancer-fighting immunity is determined by complex III of mitochondria.^272^ During differentiation of Treg cells, fusion of mitochondria triggered by transforming growth factor-beta1 (TGFβ1) is a checkpoint which directs reprogramming of metabolic processes.^273,274^ PGC1α participates in mitochondrial biogenesis and mitochondrial dynamics. Its deficiency in Tregs display attenuated suppressive functions in vivo and in vitro.^275,276^ Evidences from these studies suggest that mitochondrial dynamics are closely regulated in Tregs, which allows for precise coordination in anti-tumor immunity.

At present, the investigation into the crosstalk between immune cells and mitochondrial dynamics, like dendritic cells, neutrophils, and B cells, within the TME is lacking. Thus, there is a significant knowledge gap in terms of the interrelationship between mitochondrial dynamics and these immune cells, which are integral components of the tumor immune microenvironment. The further investigation of this field will be necessary for gaining a complete knowledge of the complex mechanisms underlying tumor progression and immune evasion, and to develop effective therapeutic strategies for cancer treatment.

## Strategies for targeting mitochondrial dynamics

In recognition of the crucial contribution of mitochondrial function and structure in cell biology, numerous disease model systems have focused on manipulating mitochondrial dynamics. Researchers have shown that restoring balances of mitochondrial dynamics by using both pharmacological and genetic approaches can enhance tissue function and increase lifespan in an animal model.^167^ Various methods have been employed to achieve this, including genetic therapies which modify gene expression to affect the division and merging of mitochondrial proteins, and chemical therapeutics which target different mechanisms crucial to mitochondrial division and merging, such as enzyme activities, interactions between proteins, and modifications post-translationally. (Table 2).

**Table 2:** Compounds and gene Intervention regulate mitochondrial dynamics for diseases

Compounds	Mechanism	Diseases	References
**Mitochondrial Fission**			
Mdivi-1	Inhibited Drp1	AD、PD、IRI	^284–286^
P110	Inhibited GTPase activity of Drp1 and its interaction with Fis1	PD、HD	^279,290^
Dynasore	Inhibited GTPase activity of Dynamin 1, Dynamin 2, Drp1	AD、CVD	^291,292^
1H-pyrrole-2-carboxamide compounds	Inhibited GTPase activity of Drp1	AD	^296^
Exenatide	Inhibited the mitochondrial localization of Drp1	HF	^294^
**Mitochondrial Fusion**			
M1	Stimulated Mitofusins	IRI	^300^
Enzyme (HO-1)	Upregulated MFN1/2 expression	Cardiomyopathy	^297^
Melatonin	Activated the Notch1/MFN2 signaling pathway, upregulated MFN2 expression	IRI	^219^
15-Oxospiramilactone (S3)	Deubiquitinated MFN1/2, augmented the activity of MFN1/2	IRI	^320^
Punicalagin	Stimulated OPA1	Diabetes	^302^
κ-opioid receptor	Stimulated OPA1	IRI	^303^
Paeonol	Stimulated OPA1	Diabetes	^230^
Gene Intervention			
Overexpression of OPA1	Promoted mitochondrial fusion by increasing expression of OPA1	AD、CVD	^264,304^
Overexpression of MFN2	Promoted fusion by increasing expression of MFN2	Cancer、Diabetes	^236,305^
Knockdown or siRNA of Drp1	Inhibited Drp1 and promotes mitochondrial fusion	Cancer	^237^

*AD* Alzheimer’s disease, *PD* Parkinson’s disease, *HF* Heart failure, *CVD* Cardiovascular diseases, *IRI* Ischemia-Reperfusion Injury, *HD* Huntington’s disease

### Compounds regulating mitochondrial dynamics

#### Regulating mitochondrial fission

Various diseases are characterized by excessive fragmentation of mitochondria and reduced fusion. To address this, fission inhibitors have been developed to decrease levels of Drp1, suppress mitochondrial division, and promote mitochondrial fusion. These inhibitors show promise in alleviating the onset and progression of disease. In recent years, research has made significant strides towards the discovery and formulation of effective fission inhibitors for mitochondria. Notable among them are Mdivi-1,^277^ P110^278^, Dynasore^279^ and DRP1i27.

Mdivi-1 is the initial inhibitor that specifically targets mitochondrial division proteins, selectively blocking the function of Drps by interacting primarily with an orthosteric domain, which is not intended to only affect GTPase domains.^277^ Mdivi-1 can correct mitochondrial shape in models of disease characterized by excess mitochondrial fission. For instance, for neuronal cells, increased research findings suggest that the compound Mdivi-1 may be a potential pharmacological agent to reduce the death of neurons due to neural degenerative disorders. Mdivi-1 treatment inhibits both apoptosis and mitophagy in genetic and environmental models of PD.^280,281^ Similarly, Mdivi-1 is capable of restoring the equilibrium of mitochondrial dynamics, and alleviating mitochondrial dysfunction correlates to an increase in autophagy triggered by Aβ in neuronal models for AD.^282^ Moreover, treatment with Mdivi-1 markedly improves behavior outcomes and reduces neurodegeneration in animal models of AD and PD.^283,284^ Besides, Mdivi-1 prevents excessive cellular necrosis in cardiomyocytes during IRI as well.^285^ In addition, Mdivi-1 prevents the development of diabetes by reducing oxidative stress and avoiding diabetes-related cardiovascular damage.^286^ Furthermore, it is also effective in preventing the reproduction and spread of cancerous cells,^246^ reversing resistance to tumor therapy,^253^ as well as enhancing MHC-I expression in mouse tumor models.^287^ Together, Mdivi-1 is a potentially new treatment for a wide range of disease states with aberrant mitochondrial fission.^288^

As a small peptide, P110 specifically inhibits the communication between Fis1 and Drp1, suppresses mitochondrial fission process.^278^ Like Mdivi-1, P110 inhibits mitochondrial division and improves performance in various neurodegenerative disease models. In particular, P110 has been found to diminish division of mitochondria and necrosis in neurons derived from patients with HD and PD.^278,289^ Dynasore is discovered from a screening of 16,000 small molecules and is the initial inhibitor of mitochondrial fission.^279^ It does not exhibit selectivity in inhibiting mitochondrial fission proteins, but affects the GTPase activities of Drp1 and Dynamin 1/2 in vitro.^279^ Studies have demonstrated that Dynasore attenuates cardiac disease and reduces neuronal damage caused by degenerative diseases *via* inhibiting excessive mitochondrial division.^290,291^

DRP1i27, a potent inhibitor of human Drp1, interacts with the GTPase domain within Drp1 by forming a hydrogen bond with Asp218 and Gln34.^292^ In fused mitochondria, DRP1i27 appears to exert dose-dependent effects, whereas Drp1-deficient cells are not affected. Moreover, DRP1i27 treatment suppresses mitochondrial fission and protects against simulated IRI.^292^

Several other small molecules have been reported to have therapeutic potential in various diseases by inhibiting Drp1 and reducing mitochondrial fission. Some of these include exenatide, which belongs to the family of glucagon-like peptides, can contribute to the improvement of heart failure.^293^ The inhibiting influence of this compound on mitochondrial division is due to its phosphorylation of Ser-637 in Drp1, which disrupts the localization of Drp1 within the mitochondria.^294^ ^1^H-pyrrole-2-carboxamide compounds have been found to improve AD symptoms.^295^ These newly identified molecules have demonstrated potential in preclinical research, but additional study is needed to evaluate their safety and efficacy in humans. Nevertheless, their discovery has opened new avenues in the search for new therapeutic approaches that target mitochondrial fission and related diseases.

#### Regulating mitochondrial fusion

Chemical therapeutics have shown promising results in restoring abnormal mitochondrial dynamics by activating and regulating mitochondrial fusion machinery. However, while mitochondrial fission inhibitors have been extensively studied, there is a lack of reported small molecule compounds that directly impact mitochondrial fusion proteins.

In mice with dilated cardiomyopathy, heme oxygenase-1 (HO-1) overexpression increased mitochondrial fusion by upregulating MFN1/2 expression.^296^ Melatonin is another MFN-promoting agent. The chemical activates the Notch1/MFN2 pathway, increasing the expression of MFN2.^219,297^ Hydrazone M1 is a compound that promotes MFN by restoring fusion of mitochondria in MFN1/2 knockout MEFs.^298^ Although the mechanism behind its action remains unclear, hydrazone M1 treatment appears to shield cells from apoptosis and modulate components of the ATP synthase complex.^298,299^ 15-oxospiramilactone (S3), a derivative of diterpenoid, is capable of targeting deubiquitinase USP30 present in mitochondria. USP30 is an isopeptidase that governs mitochondrial shape by deubiquitinating MFN1/2. Thus, S3 can amplify ubiquitination without degradation of MFN1/2, thereby eventually augments the activity of MFN1/2 and triggers mitochondrial fusion.^300^

In terms of promoting Opa1-mediated mitochondrial fusion, there are also various options. Punicalagin prevents cardiomyopathy caused by diabetes by regulating the STAT3-PTP1B pathway, thereby promoting mitochondrial fusion mediated by Opa1.^301^ Activating the κ-opioid receptor is another way to promote the fusion of mitochondria through the OPA1-STAT3 pathway, which improves cardiac adaptation to IRI.^302^ Moreover, paeonol is a compound that enhances mitochondrial fusion mediated by Opa1 through activation of the STAT3-CK2α pathway in cardiomyopathy due to diabetes.^230^

### Gene intervention alters mitochondrial dynamics

#### Overexpressing genes of mitochondrial dynamic proteins

Across both in vivo and in vitro prion disease models, reduced Opa1 expression triggered structural damage in mitochondria and neuronal apoptosis.^303^ On the other hand, overexpression of Opa1 helps to mitigate prion-induced fragmentation of mitochondrial networks, loss of mitochondrial DNA, and prevents neuron apoptosis.^303^ Moreover, Opa1 overexpression normalizes the quality control of mitochondria and maintains the function of cardiomyocytes in a model of cardiomyocyte damage induced by hypoxia.^263^ An improper balance in the level of MFN2/Drp1 leads to mitochondrial division in lung cancer cells, but when MFN2 is overexpressed, cancer cell proliferation significantly decreases, and the spontaneous apoptosis is enhanced.^236^ In individuals with NASH, MFN2 levels are significantly reduced. However, MFN2 overexpression in HepG2 cells significantly reduces ROS production and mitigates insulin resistance.^304^

Exhaustion of chimeric antigen receptor (CAR)-T cells is associated with the metabolic and mitochondrial dysfunction. Nowadays, increasing evidences have proven the significance of the mitochondria function and the metabolic status of CAR-T cells before they are infused into patients.^305,306^ In patients with complete remissions of chronic lymphocytic leukemia, CD8+ CAR T cells exhibit a greater mass of mitochondria and FAO/OXPHOS compared to cytotoxic T cell of unresponsive patients. Moreover, enhanced mitochondrial biogenesis and function positively correlated to CAR-T cell expansion and maintenance. Bezafibrate, a complex agonist of PPAR/PGC-1α, can facilitate function and fission of mitochondria in cells lacking DNM1L.^307^ It has been reported that bezafibrate can enhance OXPHOS and glycolysis of mitochondria in CTL, as well as promote naïve T cells proliferation and function. In addition, the enhancement of FAO and PPAR-α signal partially maintain the activation of CD8^+^ T cells under hypoxia and hypoglycemia.^308^ Multiple studies suggest that the reprogramming of mitochondria and metabolism during T cell expansion could potentially enhance therapeutic outcomes.^309–311^ Hence, introducing transcription factors (PGC-1α/PPARγ) or small molecules targeting mitochondrial dynamics can enhance the mitochondrial function and metabolic ability of CAR-T cells. This can potentially improve their anti-tumor efficacy in certain solid malignancies.

#### Knockdown or siRNA genes of dynamic proteins

Knockdown Drp1 promotes the development of mitochondria with elongated shapes in colon cancer cells SW480 and HCT116.^312^ An alteration in the morphology of mitochondria from fragmented granular structure in multipotential stem cell to filamentous reticular networks is required for the differentiation of cardiac mesoderm. Targeting Drp1 may be a potential strategy to stimulate human iPSCs differentiation towards heart cells for individualized regenerative cardiology.^313^ In glioma tissues, protein expression levels of Drp1 are significantly increased compared to normal brain tissues. Down-regulating Drp1 decreases invasion and proliferation of cells and inhibits the formation of pseudopodias and microvillis.^314^ Blocking Drp1 leads to compromised mitochondrial autophagy and augmented apoptosis of HCC cells during hypoxia, leading to decreased membrane potential of mitochondria as well as the release of Cyt C and apoptosis-inducing factor. Therefore, in hypoxia, inhibiting mitochondrial division and mitophagy mediated by Drp1 can increase apoptotic rates in HCC cells.^315^

## Prospects and remarks

In this review, we present characteristics and machinery of mitochondrial dynamics, the impact of mitochondrial dynamics on mitochondrial and cellular function, describe the alterations of mitochondrial dynamics in health and diseases, and provide new insights for targeting modulation of mitochondrial dynamics. However, there are still many problems to be solved in this area, and more research needs to be devoted.

Mitochondria are hubs of metabolism, as well as organelles of cellular signaling production and transmission. Mitochondrial dynamics is an adaptive change to response to the complex environments or perform specific functions.^79^ However, the molecular mechanism and regulatory signal underlying these fission-fusion balance remain largely unknown, especially for certain immune cell types. For instance, accumulation of depolarized mitochondria characterized by loss of mitochondrial mass disrupted membrane structures, which caused by decreased mitophagy activity, link with epigenetic programs toward terminal exhaustion in exhausted tumor-infiltrating CD8^+^ T cells.^316^

Since mitochondrial dynamics is an adaptive change, hindering mitochondrial morphological remodeling by interventions may impair the ability of cell adapt to the environment, even cause apoptosis and cell death.^317^ For example, TAMs always possess longer mitochondria, higher membrane potential and more ATP production per unit of glucose compared with normal macrophages, and these mitochondrial morphological changes help them to survive and perform functions in nutrient-deprived and hypoxic TME.^318^ Whether conversion TAMs into glycolysis-dependent M1-type macrophages by induction of mitochondrial fission will causes their losing the metabolic adaptation to the TME and even death?

Mitochondrial dynamics are essential for almost all living cells, and deletion of fission or fusion proteins causes lethal consequences.^79^ For instance, deletion of Drp1 or Opa1 leads to the death of mouse embryos;^319^ application of kinetic regulatory protein inhibitors (e.g. Mdivi-1) for indiscriminate inhibition of mitochondrial fission may cause cardiovascular disease.^320^ Hence, in order to precisely modulate mitochondrial dynamics, it is necessary to identify specific targets that regulate the process of mitochondrial dynamics.

Furthermore, mitochondrial dynamics exert a profound influence on cellular metabolism.^79^ In turn, whether metabolites can affect mitochondrial fission and fusion is unknown. In addition, whether there are cross-links between mitochondrial dynamics and other organs, and what are the pathways that transmit messages between them remain unclear.

In conclusion, maintaining cell and body homeostasis by adjusting mitochondrial dynamics is a promising strategy. With the advancement of research in this field, targeting mitochondrial dynamics can be an effective treatment for various diseases with mitochondrial disorders and can also improve overall health.
